# Optimizing 16S rRNA gene profile analysis from low biomass nasopharyngeal and induced sputum specimens

**DOI:** 10.1186/s12866-020-01795-7

**Published:** 2020-05-12

**Authors:** Shantelle Claassen-Weitz, Sugnet Gardner-Lubbe, Kilaza S. Mwaikono, Elloise du Toit, Heather J. Zar, Mark P. Nicol

**Affiliations:** 1 Division of Medical Microbiology, Department of Pathology, Faculty of Health Sciences, University of Cape Town, Cape Town, South Africa; 2 Department of Statistics and Actuarial Science, Faculty of Economic and Management Sciences, Stellenbosch University, Stellenbosch, South Africa; 3 Computational Biology Group and H3ABioNet, Department of Integrative Biomedical Sciences, University of Cape Town, Cape Town, South Africa; 4 Department of Science and Laboratory Technology, Dar es Salaam Institute of Technology, Dar es Salaam, Tanzania; 5 Department of Paediatrics and Child Health, Red Cross War Memorial Children’s Hospital, Cape Town, South Africa; 6 SAMRC Unit on Child & Adolescent Health, University of Cape Town, Cape Town, South Africa; 7 Institute of Infectious Disease and Molecular Medicine, Faculty of Health Sciences, University of Cape Town, Cape Town, South Africa; 8 Division of Infection and Immunity, School of Biomedical Sciences, University of Western Australia, Perth, Australia

**Keywords:** 16S rRNA gene, Bacteriome, Contamination, High-throughput sequencing, Low biomass, Mock controls, Negative controls, Optimization, Reproducibility, Respiratory

## Abstract

**Background:**

Careful consideration of experimental artefacts is required in order to successfully apply high-throughput 16S ribosomal ribonucleic acid (rRNA) gene sequencing technology. Here we introduce experimental design, quality control and “denoising” approaches for sequencing low biomass specimens.

**Results:**

We found that bacterial biomass is a key driver of 16S rRNA gene sequencing profiles generated from bacterial mock communities and that the use of different deoxyribonucleic acid (DNA) extraction methods [DSP Virus/Pathogen Mini Kit® (Kit-QS) and ZymoBIOMICS DNA Miniprep Kit (Kit-ZB)] and storage buffers [PrimeStore® Molecular Transport medium (Primestore) and Skim-milk, Tryptone, Glucose and Glycerol (STGG)] further influence these profiles. Kit-QS better represented hard-to-lyse bacteria from bacterial mock communities compared to Kit-ZB. Primestore storage buffer yielded lower levels of background operational taxonomic units (OTUs) from low biomass bacterial mock community controls compared to STGG. In addition to bacterial mock community controls, we used technical repeats (nasopharyngeal and induced sputum processed in duplicate, triplicate or quadruplicate) to further evaluate the effect of specimen biomass and participant age at specimen collection on resultant sequencing profiles. We observed a positive correlation (*r* = 0.16) between specimen biomass and participant age at specimen collection: low biomass technical repeats (represented by < 500 16S rRNA gene copies/μl) were primarily collected at < 14 days of age. We found that low biomass technical repeats also produced higher alpha diversities (*r* = − 0.28); 16S rRNA gene profiles similar to no template controls (Primestore); and reduced sequencing reproducibility. Finally, we show that the use of statistical tools for in silico contaminant identification, as implemented through the *decontam* package in R, provides better representations of indigenous bacteria following decontamination.

**Conclusions:**

We provide insight into experimental design, quality control steps and “denoising” approaches for 16S rRNA gene high-throughput sequencing of low biomass specimens. We highlight the need for careful assessment of DNA extraction methods and storage buffers; sequence quality and reproducibility; and in silico identification of contaminant profiles in order to avoid spurious results.

## Background

High-throughput 16S ribosomal ribonucleic acid (rRNA) gene sequencing has the potential to provide detailed characterization of microbial communities from a range of ecological niches in humans [1–3]. These range from the densely colonized gastro-intestinal tract [4, 5] to low biomass sites including the lower respiratory tract [6–8] and womb [9, 10] which have previously been considered sterile. However, there is much controversy surrounding evidence from 16S rRNA sequencing studies supporting opposing hypotheses on womb sterility - in particular the placental microbiome [11, 12] - and on the accuracy of studies of low biomass samples more broadly [13, 14].

**Fig. 1:**
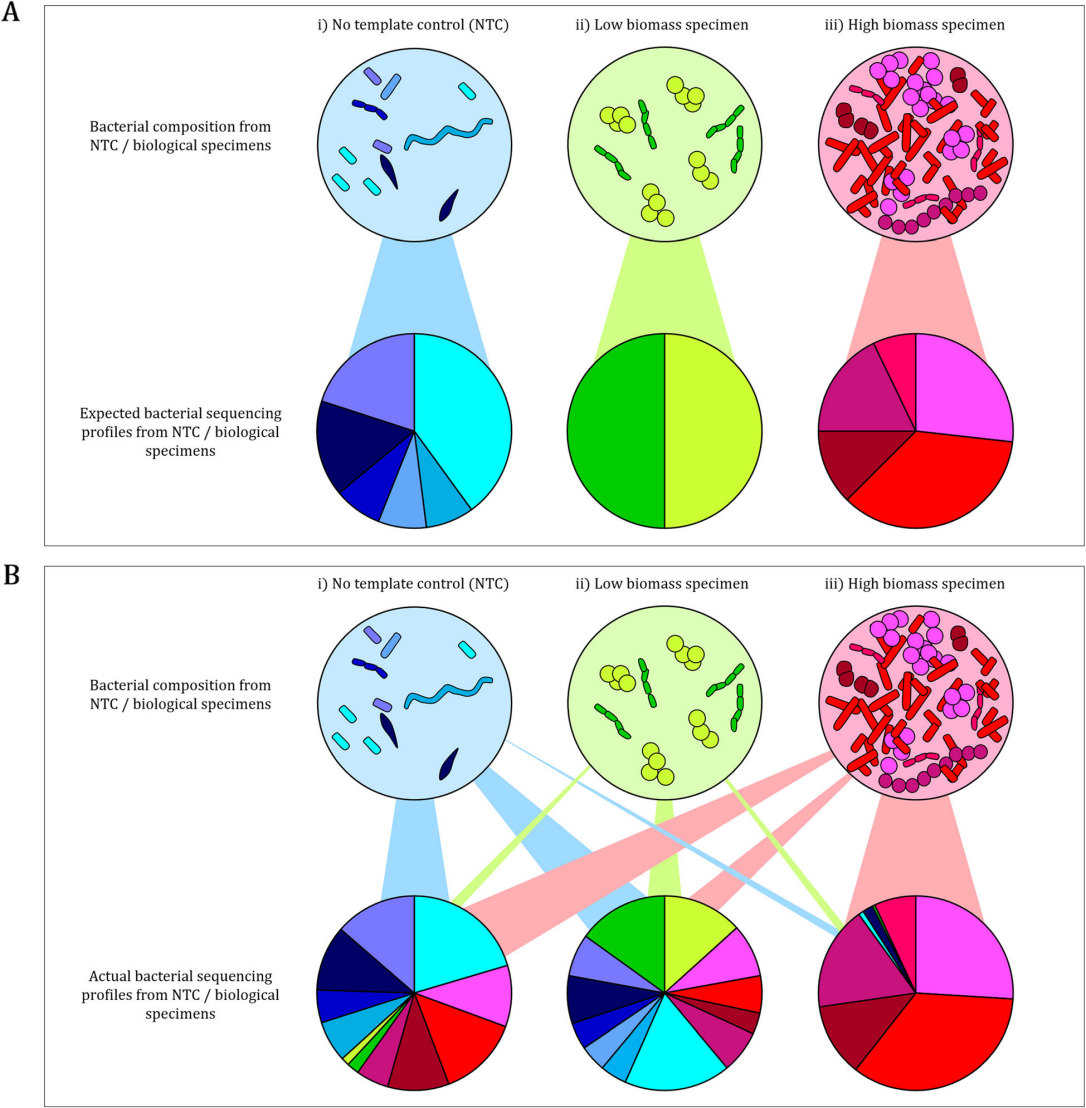
Representation of **a** expected and **b** actual sequencing profiles from no template controls, low biomass and high biomass specimens following 16S rRNA gene sequencing. **a** Expected 16S rRNA gene sequencing profiles from i) no template controls (NTCs), ii) low biomass and iii) high biomass biological specimens which corresponds with their endogenous bacterial composition. **b** Actual 16S rRNA gene sequencing profiles generated from i) NTCs may comprise of reagent and laboratory contaminants as well as exogenous sequences from low and high biomass specimens (well-to-well contamination); ii) low biomass biological specimen sequencing profiles may be overrepresented by exogenous profiles from both NTCs (reagent and laboratory contaminants) and high biomass specimens (well-to-well contamination); whilst iii) high biomass sequencing profiles are expected to be least affected by reagent and laboratory contaminants present in NTCs and cross-contamination from low biomass specimens

A primary concern when using 16S rRNA gene sequencing to analyse specimens with low levels of endogenous deoxyribonucleic acid (DNA) is preferential amplification and sequencing of contaminant DNA originating from reagents or the laboratory environment [15–18]. No template controls (NTCs) such as storage buffers, elution buffers, or water may serve as good approximates for “contaminants” introduced during extraction and library preparation steps [16, 18, 19] (Fig. 1a). However, DNA and amplicon “spill-over” from high biomass to low biomass specimens (also referred to as well-to-well contamination) adds to exogenous biological profiles in neighbouring low biomass specimens [20] (Fig. 1b). This physical exchange of DNA/amplicon between biological specimens, and between biological specimens and NTCs (Fig. 1b), calls for a rigorous in silico approach to identify and remove contamination. For example, simply subtracting NTC contaminant profiles from biological specimens introduces the risk of removing true biological profiles alongside contaminant profiles [18] (Fig. 1b). Some studies have used in silico approaches to remove previously reported potential contaminant profiles [21, 22], however, contaminant profiles are likely to vary between laboratories and between experiments within laboratories. Hence, the only way to efficiently deal with contaminants is to include controls tailored to each experiment and to use optimal decontamination approaches.

In addition to addressing contamination, 16S rRNA gene sequencing studies also need to validate the process of DNA extraction and polymerase chain reaction (PCR) amplification, which is typically done via the inclusion of bacterial mock communities as extraction and sequencing controls [23]. These mixtures of known bacterial composition can be generated to mimic biological specimens and used to identify optimal DNA extraction methods for the specimen type of interest. Due to their known composition, they are also used to evaluate sequencing reproducibility and identify contaminants. However, mock community controls also need to mimic biological profiles in their biomass, as specimen biomass contributes to the level of contaminants and sequencing reproducibility observed in any given sequencing run. Therefore, biological specimens - with inherent variation in biomass - randomly selected for repeat extraction and/or sequencing within and between different sequencing runs may provide an added benefit when measuring reproducibility and identifying potential contaminants.

Standardization of bacterial profiling methods provides opportunity for multicentre comparative analyses [24], however, this may not rule out technical biases. Protocols for analysing bacterial profiles from low biomass specimens, which are at particular risk of PCR bias and contamination, need to be optimized prior to standardization. Nasopharyngeal (NP) specimens are one such example as they have bacterial densities of < 10^6^ 16S copies/mL [15, 17, 25]. In order to contribute to the development of robust 16S rRNA gene sequencing protocols for low biomass specimens, we outline important quality control steps using NP and induced sputum (IS) specimens collected from infants. Our first objective was to determine how different DNA extraction methods, bacterial biomass and specimen storage buffers could influence 16S rRNA gene sequencing profiles from bacterial mock community controls. Our second objective was to investigate whether specific characteristics from low biomass biological specimens (NP and IS) correlates with sequencing quality. Finally, our third objective was to evaluate the use of two methods for in silico identification of potential contaminants from 16S rRNA gene sequencing data generated from low biomass biological specimens (NP and IS). Overall, we aimed to provide additional insight into experimental design, quality control steps and “denoising” approaches for sequencing low biomass specimens.

## Results

### DNA extraction method, specimen biomass and storage buffer influence 16S rRNA gene sequencing profiles

This section describes sequencing profiles generated using DNA extracts from high and low biomass bacterial mock community controls (Zymobiomics-Primestore-high, Zymobiomics-STGG-high, Zymobiomics-Primestore-low and Zymobiomics-STGG-low) generated using a commercial bacterial mock community (Zymobiomics-Cells) and two storage buffers (Primestore and STGG) (Table 1, Additional file 1). For each of the four bacterial mock communities, we present sequencing profiles generated from triplicate DNA extracts using two DNA extraction kits (Kit-QS and Kit-ZB) (Table 2). In addition, we report on sequencing profiles from two pre-extracted commercially available bacterial mock community DNA controls (Zymobiomics-DNA and BEI-DNA), included as sequencing controls (Table 1, Table 2).

**Table 1:**
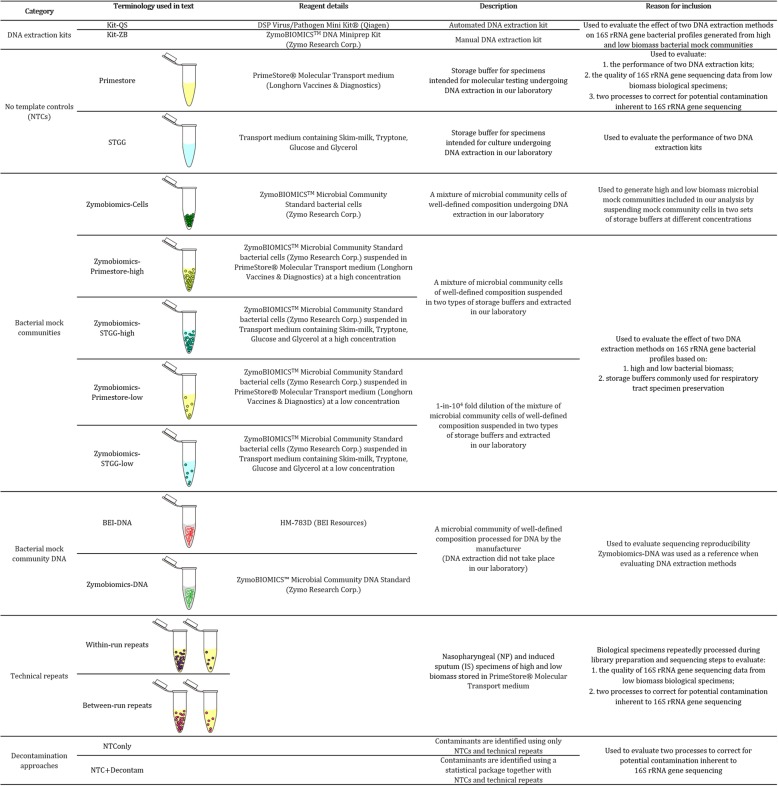
Reference guide to DNA extraction kits, storage buffers/no template controls, bacterial mock communities, technical repeats and decontamination approaches

**Table 2:** Extraction and sequencing controls included in this study

Control type	Control subtype	Control name	Composition	Source	DNA extraction performed in our laboratory	DNA extraction kit	Extraction replicates	Sequencing replicates	Total included for equencing
Extraction controls	Bacterial mock communities	Zymobiomics-Primestore-high	900 μl of Zymobiomics-Cells suspended in 3600 μl Primestore	Zymo Research Corp., Irvine, CA, United States & Longhorn Vaccines & Diagnostics, Bethesda, MD, USA	✓	Kit-QS Kit-ZB	3 per DNA extraction kit	–	6
		Zymobiomics-STGG-high	900 μl of Zymobiomics-Cells suspended in 3600 μl STGG	Zymo Research Corp., Irvine, CA, United States & National Health Laboratory Services, Cape Town, South Africa	✓	Kit-QS Kit-ZB	3 per DNA extraction kit	–	6
		Zymobiomics-Primestore-low	1-in-10^4^ fold dilution of Zymobiomics-Primestore-high	Zymo Research Corp., Irvine, CA, United States & Longhorn Vaccines & Diagnostics, Bethesda, MD, USA	✓	Kit-QS Kit-ZB	3 per DNA extraction kit	–	6
		Zymobiomics-STGG-low	1-in-10^4^ fold dilution of Zymobiomics-STGG-high	Zymo Research Corp., Irvine, CA, United States & National Health Laboratory Services, Cape Town, South Africa	✓	Kit-QS Kit-ZB	3 per DNA extraction kit	–	6
	NTCs	Primestore	Storage buffer Primestore	Longhorn Vaccines & Diagnostics, Bethesda, MD, USA	✓	Kit-QS Kit-ZB	3 per DNA extraction kit	–	6
		STGG	Storage buffer STGG	National Health Laboratory Services, Cape Town, South Africa	✓	Kit-QS Kit-ZB	3 per DNA extraction kit	–	6
Sequencing controls	Bacterial mock community DNA	BEI-DNA	1-in-10 fold dilution of HM-783D in Milli-Q® ultrapure water	BEI Resources, NIAID, NIH as part of the Human Microbiome Project, Manassas, VA, USA & MilliporeSigma, Burlington, MA, USA	x	Not specified	–	–	3
		Zymobiomics-DNA	1-in-10 fold dilution of ZymoBIOMICS™ Microbial Community DNA Standard in Milli-Q® ultrapure water	Zymo Research Corp., Irvine, CA, United States & MilliporeSigma, Burlington, MA, USA	x	Not specified	–	–	8
	Technical repeats	Within-run repeats	NP and IS specimens stored in Primestore	Collected from infants enrolled in the DCHS [26]	✓	Kit-QS	–	2 per specimen	86
		Between-run repeats	NP and IS specimens stored in Primestore	Collected from infants enrolled in the DCHS [26]	✓	Kit-QS	–	2, 3 or 4 per specimen	123
	NTCs	Primestore	Storage buffer Primestore	Longhorn Vaccines & Diagnostics, Bethesda, MD, USA	✓	Kit-QS	–	–	35

*Zymobiomics-Cells* ZymoBIOMICS™ Microbial Community Standard bacterial cells (Catalog No. D6300, Zymo Research Corp., Irvine, CA, United States)*, Kit-QS* DSP Virus/Pathogen Mini Kit® using QIAsymphony® SP instrument (catalogue no. 937036, Qiagen GmbH, Hilden, Germany), *Kit-ZB* ZymoBIOMICS DNA Miniprep Kit (catalogue no. ZR D4300, Zymo Research Corp., Irvine, CA, United States)*, NTCs* No template controls*, Primestore* PrimeStore® Molecular Transport medium (Longhorn Vaccines & Diagnostics Bethesda, MD, USA), *STGG* transport medium containing Skim-milk*,* Tryptone, Glucose and Glycerol*, NP* Nasopharyngeal swabs *IS* Induced sputum*, DCHS* Drakenstein Child Health Study

Bacterial profiles sequenced from BEI-DNA controls (*n* = 3) showed good reproducibility and comparability to manufacturer’s specifications [27] (Additional file 2). Three genera, reported at low abundances by the manufacturer (*Actinomyces* 0.02%, *Deinococcus* 0.02% and *Propionibacterium* 0.2%) [27], were not detected from the DNA amplified and sequenced in our laboratory. We detected 11 additional genera (6 of which were unclassifiable at genus-level) at low abundances from BEI-DNA controls processed in our laboratory. The sequenced profiles from Zymobiomics-DNA controls (*n* = 8) also matched the manufacturer’s specifications with the exception of a few low abundant OTUs (Additional file 2). We detected 35 additional genera (16 of which were unclassifiable at genus-level) at low abundances from the eight Zymobiomics-DNA controls processed in our laboratory (Additional file 2).

Data generated from the four bacterial mock communities (Zymobiomics-Primestore-high, Zymobiomics-Primestore-low, Zymobiomics-STGG-high and Zymobiomics-STGG-low) showed that Kit-QS extracted purer DNA compared to Kit-ZB based on the ratio of absorbance (260 nm and 280 nm) measured by the NanoDrop® ND-1000 (Table 3). We only compared ratio of absorbance measures for the two kits using high biomass bacterial mock communities, as low biomass bacterial mock community dsDNA concentrations were outside the NanoDrop® ND-1000 Spectrophotometer’s lower limit of detection. As much as 100-fold more 16S rRNA gene copies per millilitre of specimen input volume was extracted from low biomass bacterial mock communities using Kit-ZB (Table 3). This observation was only made for low biomass bacterial mock communities generated using Primestore storage buffer. Bacterial profiles resulting from triplicate extractions of each of the four bacterial mock communities (Zymobiomics-Primestore-high, Zymobiomics-Primestore-low, Zymobiomics-STGG-high and Zymobiomics-STGG-low) were highly reproducible for both extraction methods: Kit-QS (*n* = 12) [bacterial mock communities coefficient of determination in linear regression analysis (R^2^): 0.96 (interquartile range (IQR): 0.94–0.98)] and Kit-ZB (*n* = 12) [bacterial mock communities R^2^: 0.98 (IQR: 0.96–0.99)].

**Table 3:** Quantity and quality of DNA extracted using two DNA extraction methods

Control	DNA extraction kit	Buffer	Replicate	16S rRNA gene copy numbers (copies/ml of specimen input volume)	260/280 NanoDrop® ND-1000 ratio
Zymobiomics-Primestore-high	Kit-QS	Primestore	1	2.47E^9^	1.68
			2	2.06E^9^	1.75
			3	1.92E^9^	1.90
	Kit-ZB	Primestore	1	2.14E^9^	2.09
			2	1.99E^9^	1.34
			3	1.61E^9^	1.19
Zymobiomics-Primestore-low	Kit-QS	Primestore	1	3.77E^3^	–
			2	5.82E^3^	–
			3	5.37E^3^	–
	Kit-ZB	Primestore	1	2.08E^5^	–
			2	2.72E^5^	–
			3	3.43E^5^	–
Zymobiomics-STGG-high	Kit-QS	STGG	1	7.52E^8^	1.96
			2	7.32E^8^	1.89
			3	5.58E^8^	1.93
	Kit-ZB	STGG	1	3.09E^9^	2.53
			2	1.89E^9^	1.34
			3	1.73E^9^	1.23
Zymobiomics-STGG-low	Kit-QS	STGG	1	2.79E^5^	–
			2	3.58E^5^	–
			3	5.10E^5^	–
	Kit-ZB	STGG	1	1.89E^5^	–
			2	2.85E^5^	–
			3	2.76E^5^	–

*Zymobiomics-Primestore-high* 900 μl of Zymobiomics-Cells suspended in 3600 μl Primestore*, Zymobiomics-Primestore-low* 1-in-10^4^ fold dilution of Zymobiomics-Primestore-high*, Zymobiomics-STGG-high,* 900 μl of Zymobiomics-Cells suspended in 3600 μl STGG*, Zymobiomics-STGG-low* 1-in-10^4^ fold dilution of Zymobiomics-STGG-high*, Kit-QS* DSP Virus/Pathogen Mini Kit® using QIAsymphony® SP instrument (catalogue no. 937036, Qiagen GmbH, Hilden, Germany)*, Kit; ZB* ZymoBIOMICS DNA Miniprep Kit (catalogue no. ZR D4300, Zymo Research Corp., Irvine, CA, United States)*, Primestore* PrimeStore® Molecular Transport medium (Longhorn Vaccines & Diagnostics Bethesda, MD, USA)*, STGG* Storage medium containing skim milk, tryptone, glucose, and glycerine

**Fig. 2:**
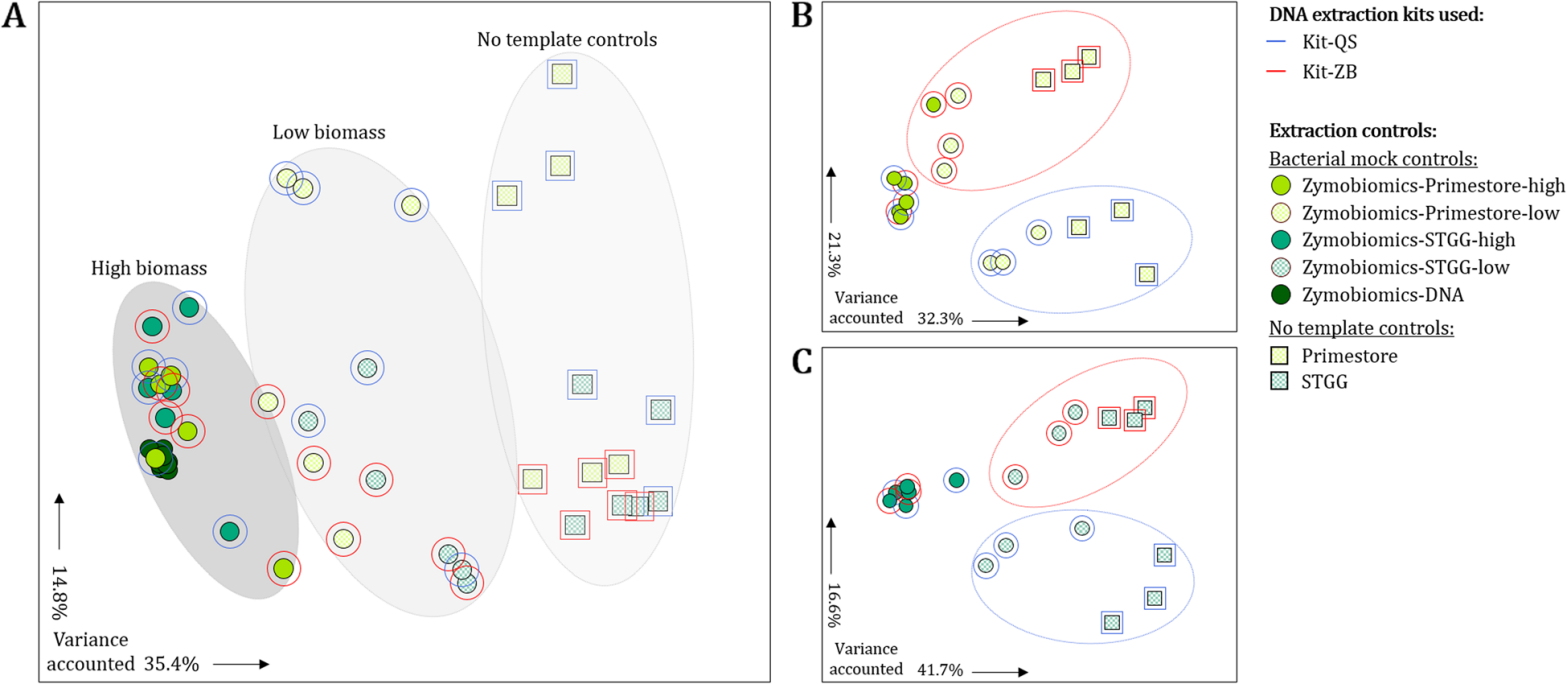
16S rRNA gene bacterial profiles are reflective of specimen biomass and are further influenced by DNA extraction methods and storage buffers. **a** Differences in beta diversities (calculated at OTU-level) measured from all bacterial mock community controls and no template controls (NTCs). **b** Differences in beta diversities measured from bacterial mock community controls and NTCs generated using Primestore storage buffer. **c** Differences in beta diversities measured from bacterial mock community controls and NTCs generated using STGG storage buffer. The proportion of variance captured by coordinate analysis axes are shown in the bottom left corner of each panel. Blue and red colours represent DNA extraction methods Kit-QS and Kit-ZB, respectively. Shades of chartreuse filled circles represent bacterial mock communities generated using Primestore storage buffer (solid-filled chartreuse circles: high biomass bacterial mock communities; pattern-filled chartreuse circles: low biomass bacterial mock communities). Shades of emerald filled circles represent bacterial mock communities generated using STGG storage buffer (solid-filled emerald circles: high biomass bacterial mock communities; pattern-filled emerald circles: low biomass bacterial mock communities). Dark green filled circles represent Zymobiomics-DNA. Chartreuse and emerald pattern-filled squares represent Primestore and STGG NTCs, respectively

Principal coordinate analysis at OTU-level showed that bacterial biomass is a key driver of 16S rRNA gene profiles (analysis of beta diversities by PERMANOVA: *P* = 0.001) (Fig. 2a). High biomass bacterial mock communities (Zymobiomics-Primestore-high and Zymobiomics-STGG-high) grouped together in a compact cluster alongside Zymobiomics-DNA, whilst low biomass bacterial mock communities (Zymobiomics-Primestore-low and Zymobiomics-STGG-low) clustered midway between their undiluted counterparts and NTCs (Primestore and STGG) (Fig. 2a). Beta diversities were also significantly different when comparing the two DNA extraction methods (*P* = 0.001) and storage buffers (*P* = 0.001) (Fig. 2a). When stratifying our analyses based on the two storage buffers [Primestore (Fig. 2b) and STGG (Fig. 2c)], we observed clear differentiation in beta diversities in relation to specimen biomass (*P* = 0.001) and the DNA extraction method (*P* = 0.001) used.

In order to assess the efficiency of the two extraction methods in extracting DNA from hard- and easy-to-lyse bacteria, we compared sequencing profiles at OTU-level from Zymobiomics-Primestore-high (*n* = 6) and Zymobiomics-STGG-high (*n* = 6) to Zymobiomics-DNA (*n* = 8). Compared to Zymobiomics-DNA, both Kit-QS and Kit-ZB yielded an over-representation of easy-to-lyse gram-negative bacteria (*Pseudomonas*, *Salmonella* and *Escherichia-Shigella*) and an under-representation of hard-to-lyse gram-positive bacteria (*Enterococcus*, *Staphylococcus*, *Listeria* and *Bacillus*) (Additional file 3). Differences between Zymobiomics-DNA and the two DNA extraction methods were more marked for Kit-ZB (Additional file 4 a). The hard-to-lyse gram-positive bacteria *Enterococcus*, *Staphylococcus* and *Listeria* were also significantly less represented by Kit-ZB when compared to Kit-QS (Additional file 4 b).

**Fig. 3:**
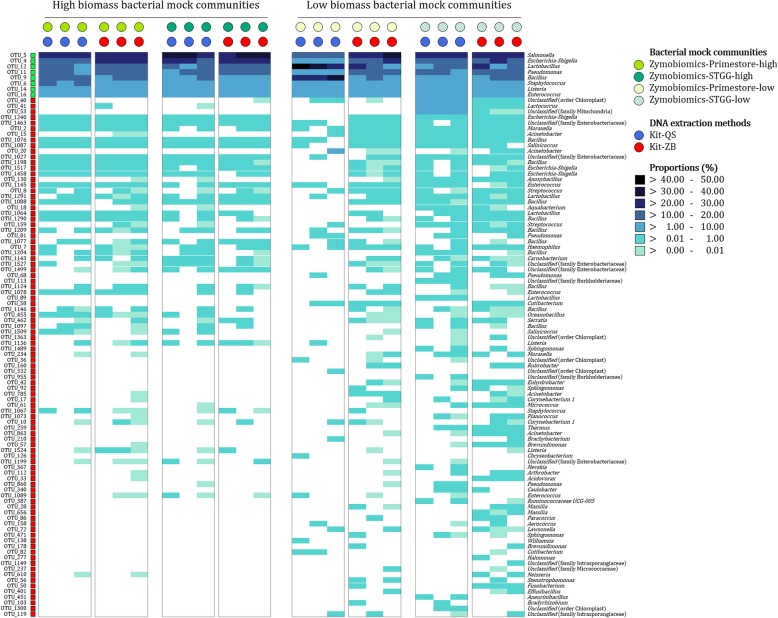
Proportions of operational taxonomic units (OTUs) in four bacterial mock communities using two DNA extraction methods, with triplicate testing. A gradient scale is used to represent the proportions of the 100 most abundant OTUs detected across the bacterial mock community controls. OTU and genus-level classifications are provided on the left and right side of the figure, respectively. OTUs expected in each of the four bacterial mock communities are shown using green squares. Red squares denote OTUs not expected in the four bacterial mock communities (“background OTUs”). The bacterial mock communities (Primestore vs STGG), their biomass (high versus low) and the DNA extraction methods used are denoted at the top of each heatmap

We detected more “background OTUs” (OTUs not expected in mock communities) from low biomass bacterial mock communities (Zymobiomics-Primestore-low and Zymobiomics-STGG-low) compared to high biomass bacterial mock communities (Zymobiomics-Primestore-high and Zymobiomics-STGG-high), irrespective of the DNA extraction method and storage buffer used (Fig. 3). The latter was more pronounced from low biomass profiles generated using storage buffer STGG (average proportion of “contaminant OTUs”: 9.5%) when compared to Primestore (average proportion of “contaminant OTUs”: 1.5%).

In summary, we show that the bacterial biomass is an important determinant of 16S rRNA gene sequencing profiles and that storage buffers and DNA extraction methods further influence these profiles. When comparing Kit-QS to Kit-ZB, we observed better quality of DNA and a better representation of hard-to-lyse bacteria from profiles generated using Kit-QS. Finally, we show that the use of different storage buffers impacts on low biomass “background OTUs”, with the commonly used STGG buffer associated with increased background.

### Quality of 16S rRNA gene sequencing data from respiratory tract specimens correlates with specimen biomass

We validated 16S rRNA gene sequencing data generated across 11 sequencing runs from low biomass technical repeats (NP and IS specimens) and determined criteria for excluding specimens based on the likelihood of spurious results. We compared 1) technical repeat profiles to NTC profiles, 2) examined sequencing reproducibility and 3) investigated the presence of “spurious OTUs” in relation to specimen biomass, participant age at specimen collection and read counts following bioinformatic processes. In total, we analysed 244 sequencing libraries generated from 35 Primestores (NTCs) and 209 technical repeats (NP and IS specimens) (Table 1, Table 2). All technical repeats had a minimum of two sequencing profiles available for analysis. We generated 86 sequencing profiles from within-run repeats (43 NP/IS specimens processed in duplicate within respective runs); and 123 sequencing profiles from between-run repeats [NP/IS specimens processed in duplicate (*n* = 30), triplicate (*n* = 9) and quadruplicate (*n* = 9) across different runs].

#### Participant age at specimen collection correlates with specimen biomass which in turn correlates with sequencing metrics (read counts, alpha diversity, OTU counts and “spurious OTUs”)

**Fig. 4:**
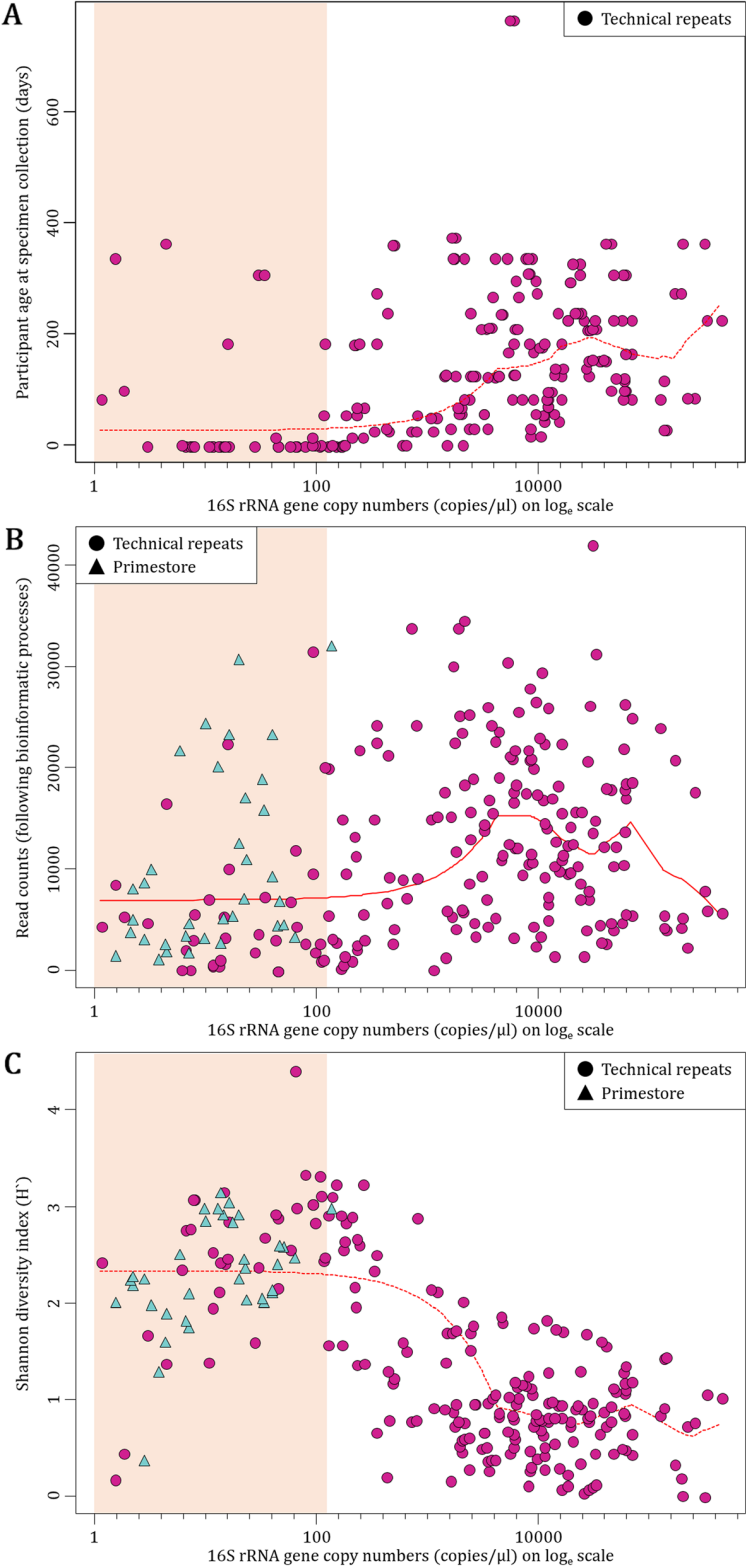
Participant age at specimen collection, read counts and alpha diversity relative to specimen biomass for no template controls (Primestore, *n* = 35) and technical repeats (*n* = 209). **a** Scatter plot of participant age at specimen collection **b** read counts following bioinformatic processes and **c** Shannon diversity indices (alpha diversity) at OTU-level in relation to specimen biomass (16S rRNA gene copies/μl) plotted on log_e_ scale. Vertical pink shaded area highlights 16S rRNA gene copies/μl < 500

We observed a positive correlation (*r* = 0.16) between 16S rRNA gene copy numbers (representing specimen biomass) and participant age at which specimens were collected (Fig. 4a). The majority (33/37; 89%) of technical repeats collected at < 1 day of life (birth specimens) had < 500 16S rRNA gene copies/μl (Fig. 4a). The majority of specimens with < 500 16S rRNA gene copies/μl (low biomass technical repeats) were collected at < 14 days of age (37/61; 61%), whilst almost all specimens with > 500 16S rRNA gene copies/μl (high biomass technical repeats) were collected at > 14 days of age (144/148; 97%) (Additional file 5).

We also observed a marginally negative correlation (*r* = − 0.04) between specimen biomass and reads available for downstream analysis (Fig. 4b). Low biomass technical repeats (< 500 16S rRNA gene copies/μl) had a median read count of 4400 (IQR: 1890–9691) compared to 13,377 (IQR: 7093–20,213) observed from high biomass technical repeats. There was an increase in the median read counts with increasing age for both low and high biomass technical repeats, respectively [< 14 days: 2815 (IQR: 989–5643) and 6610 (IQR: 5981–7680) vs. > 14 days: 8930 (IQR: 4042–17,527) and 13,897 (IQR: 7128–20,747) (Additional file 5).

In addition, specimen biomass correlated negatively (*r* = − 0.28) with alpha diversity (Shannon diversity index) [< 500 16S rRNA gene copies/μl: 2.5 (IQR: 1.7–2.9) vs. > 500 16S rRNA gene copies/μl: 0.8 (0.5–1.1)] (Fig. 4c). The median alpha diversity from low biomass technical repeats was comparable to that measured from Primestore [2.3 (IQR: 1.7–2.9) vs. 2.2 (IQR: 2.0–2.7)] (Fig. 4c). We also observed a negative correlation between specimen biomass and the number of OTUs sequenced [< 500 16S rRNA gene copies/μl: 101 (IQR: 48–142) vs. > 500 16S rRNA gene copies/μl: 42 (27–70)] (Additional file 5); and specimen biomass and the number of “spurious OTUs” (OTUs with a total of < 5 reads amongst all 244 technical repeats and Primestore) [< 500 16S rRNA gene copies/μl: 2 (IQR: 0–4) vs. > 500 16S rRNA gene copies/μl: 0 (0–1.3)]. The “per specimen frequency” at which spurious OTUs were identified (number of spurious OTUs/number of technical repeats) was 3.1 for low biomass technical repeats compared to 1.0 for high biomass technical repeats (Additional file 5).

#### Sequencing profiles from low biomass technical repeats (NP and IS specimens), collected during the first two weeks of life, are similar to profiles from no template controls (Primestore)

**Fig. 5:**
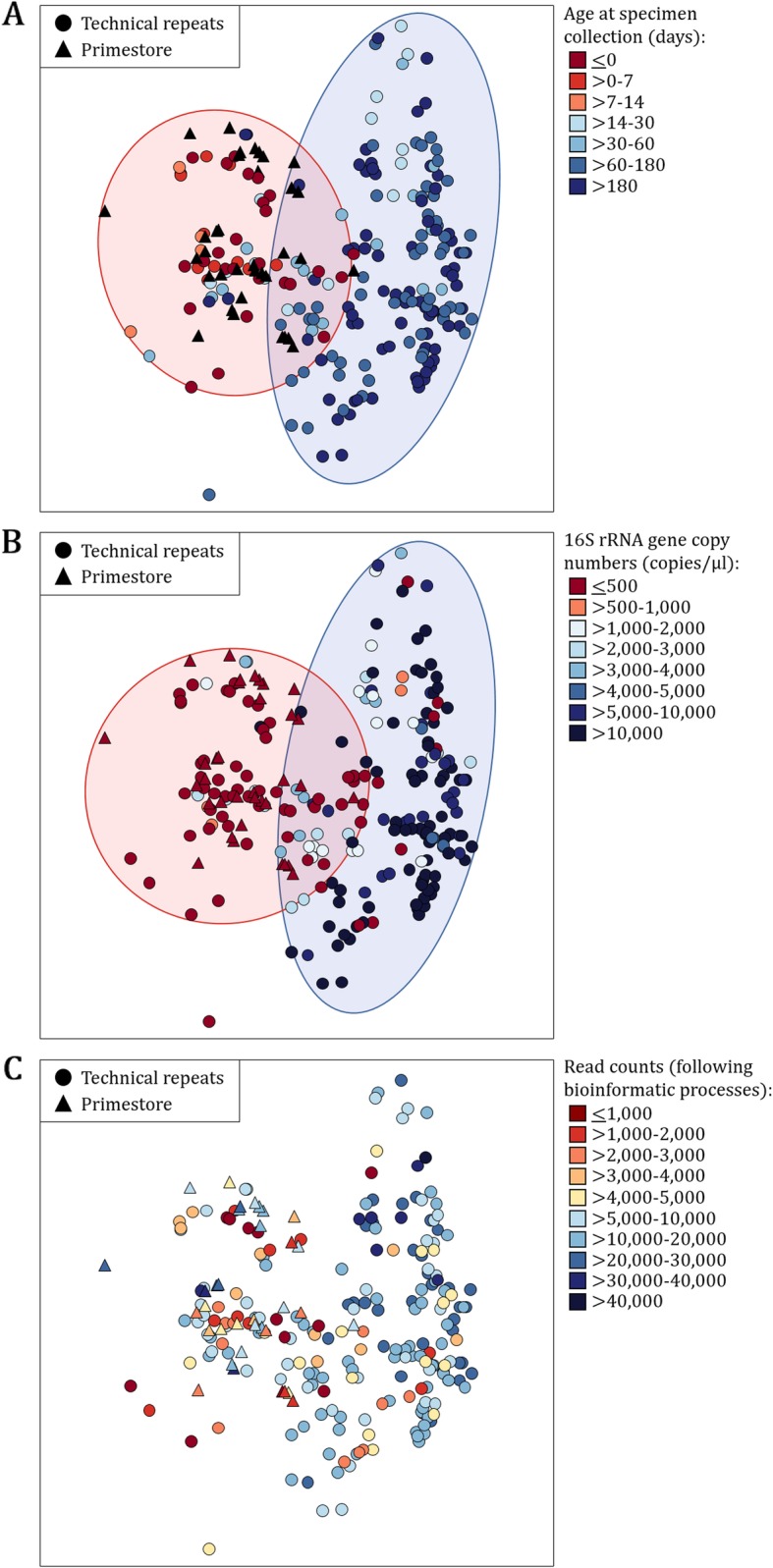
Logarithm of ratio-transformed data (log-ratio) biplots in relation to participant age at specimen collection, 16S rRNA gene copies/μl and read counts following bioinformatic processing. Data points are coloured according to **a** participant age at specimen collection (in days), **b** 16S rRNA gene copies/μl and **c** read counts available for downstream analyses. Technical repeats (*n* = 209) are represented using filled circles. No template controls (Primestore, *n* = 35) are represented using filled triangles

Using logarithm of ratio-transformed data (log-ratio) biplots, we observed two distinct clusters in relation to participant age at specimen collection (Fig. 5a) and specimen biomass (16S rRNA gene copies/μl) (Fig. 5b). Almost all technical repeats collected at < 14 days of life clustered closely with Primestore (Fig. 5a). This was also evident for a subset of specimens collected at > 14 days of life. The majority of technical repeats with < 500 16S rRNA gene copies/μl clustered with Primestore (Fig. 5b). Although less marked, we also observed a tendency of specimens with lower read counts to cluster with Primestore (Fig. 5c).

**Fig. 6:**
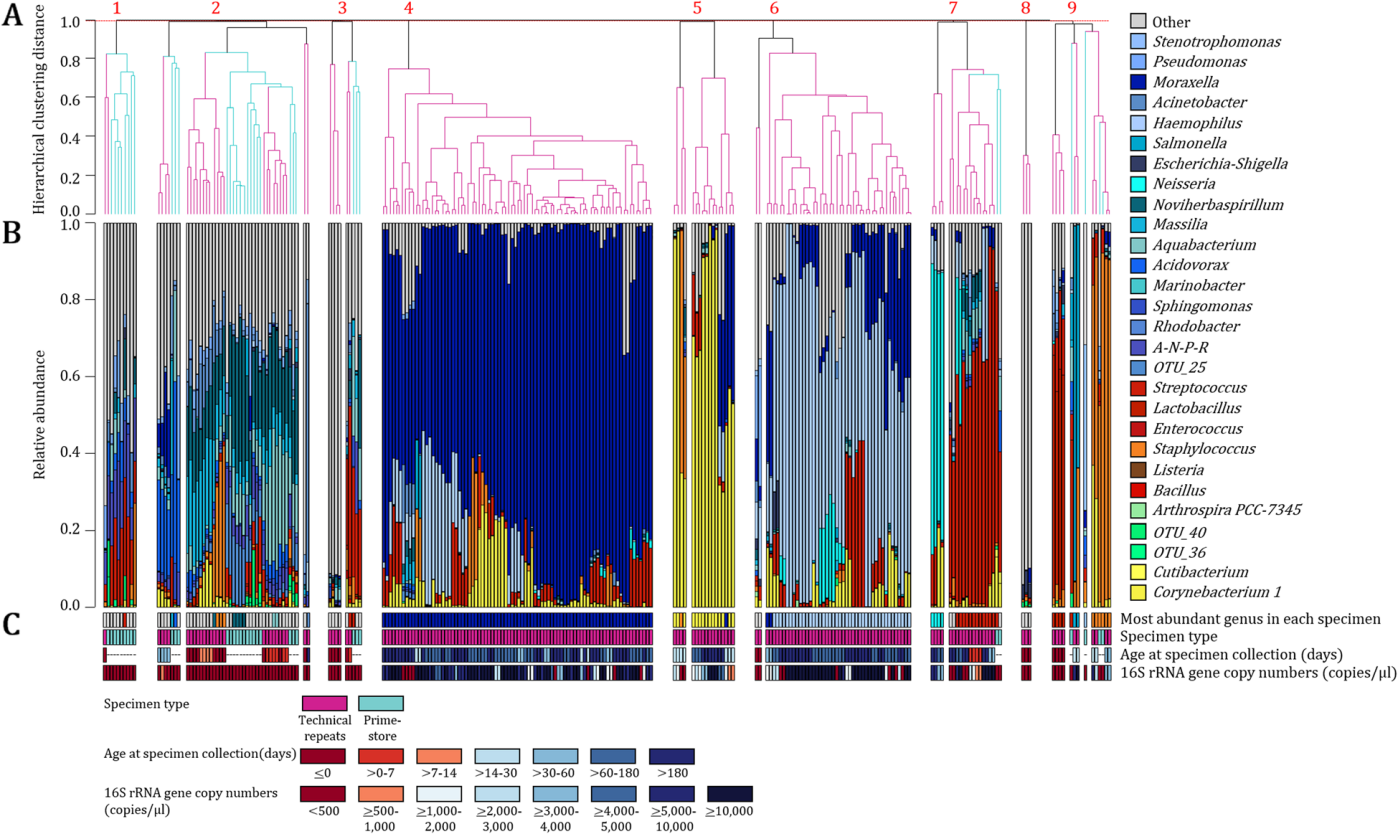
Bacterial composition in no template controls (Primestore, *n* = 35) and low biomass technical repeats (*n* = 209). **a** Dendogram representing unsupervised hierarchical clustering distances are based on Bray Curtis dissimilarity indices calculated at OTU-level. The dendogram is colour-coded based on specimen type (Primestore: darkturquoise; technical repeats: deeppink). **b** Differences between Primestore and technical repeats are shown at genus-level, with colour-codes representing phylum-level classification (Shades of blue: *Proteobacteria*, shades of red: *Firmicutes*). Genera with proportions < 1% in each of the specimens are grouped together as “Other” and shown in grey. **c** Most abundant genera within each the specimens, specimen type, participant age at specimen collection (in days) and 16S rRNA gene copy numbers (copies/μl) are summarised at the bottom of the Fig. *A-N-P-R*: *Allorhizobium-Neorhizobium-Pararhizobium-Rhizobium*

Unsupervised clustering analysis at OTU-level generated nine primary clusters (complete linkage clustering distance set to 0.99) (Fig. 6). A clustering distance set to 0.90 generated up to four sub-clusters within each of the nine primary clusters. Clusters 1–3 and 9 consisted of both Primestore and technical repeats, whilst clusters 4–8 primarily consisted of technical repeats. The majority of technical repeats in clusters 1–3, which included an even mixture of Primestore (29/62, 47%) and technical repeats (33/62, 53%), represented early life specimens (collections at < 14 days of age: 28/33, 85%) of low biomass (< 500 16S rRNA gene copies per μl: 32/33, 97%). All Primestores in clusters 1–3 were also of low biomass (< 500 16S rRNA gene copies per μl). OTUs classified as “Other” at genus-level were most abundant in Primestore and technical repeats throughout clusters 1–3. Clusters 4–6 consisted only of technical repeats, the majority of which were collected at > 14 days of age (143/145, 99%) with > 500 16S rRNA gene copies per μl (127/145, 88%). OTUs belonging to the genera *Moraxella*, *Corynebacterium* and *Haemophilus* (commonly detected from respiratory tract specimens) were most abundant in technical repeats from clusters 4, 5 and 6, respectively. Cluster 7 primarily consisted of technical repeats (18/20, 90%), primarily consisting of OTUs belonging to the genera *Neisseria* (*n* = 3) and *Streptococcus* (*n* = 15). As for clusters 4–6, technical repeats in cluster 7 were also primarily collected at > 14 days of age (14/18, 78%) with high biomass (> 500 16S rRNA gene copies per μl: 12/18, 67%), however, these were slightly less prevalent when compared to clusters 4–6 (78% vs. 99 and 67% vs. 88%, respectively). Cluster 8 consisted of only three technical repeats, all of which were collected at < 0 days of age with < 500 16S rRNA gene copies per μl. OTUs predominating these profiles were classified as “Other” at genus-level. Cluster 9 had an uneven representation of technical repeats (10/14, 71%) and Primestore (4/14, 29%). Fewer technical repeats from cluster 9 were collected at > 14 days of age with > 500 16S rRNA gene copies per μl when compared to cluster 7 and clusters 4–6 (60% vs. 78% vs. 99 and 60% vs. 67% vs. 88%, respectively). Technical repeats in cluster 9 primarily consisted of OTUs belonging to *Lactobacillus* (*n* = 4), *Salmonella* (*n* = 2) and *Staphylococcus* (*n* = 4) genera, respectively.

#### Specimen features, such as participant age at specimen collection, 16S rRNA gene copy numbers and read counts as proxy for sequencing reproducibility

We determined the reproducibility of sequenced profiles among technical repeats included in our dataset. We calculated a R^2^ value for each of the duplicate sequencing profiles (*n* = 73) present in our dataset, three R^2^ values for each of the triplicate sequencing profiles (*n* = 27), and six R^2^ values for each of the quadruplicate sequencing profiles (*n* = 54). Overall, sequencing reproducibility was high across the entire dataset [median R^2^ = 0.992 (IQR: 0.951–0.999)]. Sequencing profiles generated from within-run technical repeats (*n* = 86) were more reproducible compared to those from between-run repeats (*n* = 123) [median R^2^ = 0.999 (IQR: 0.991–1.000) vs 0.982 (IQR: 0.942–0.997)].

**Fig. 7:**
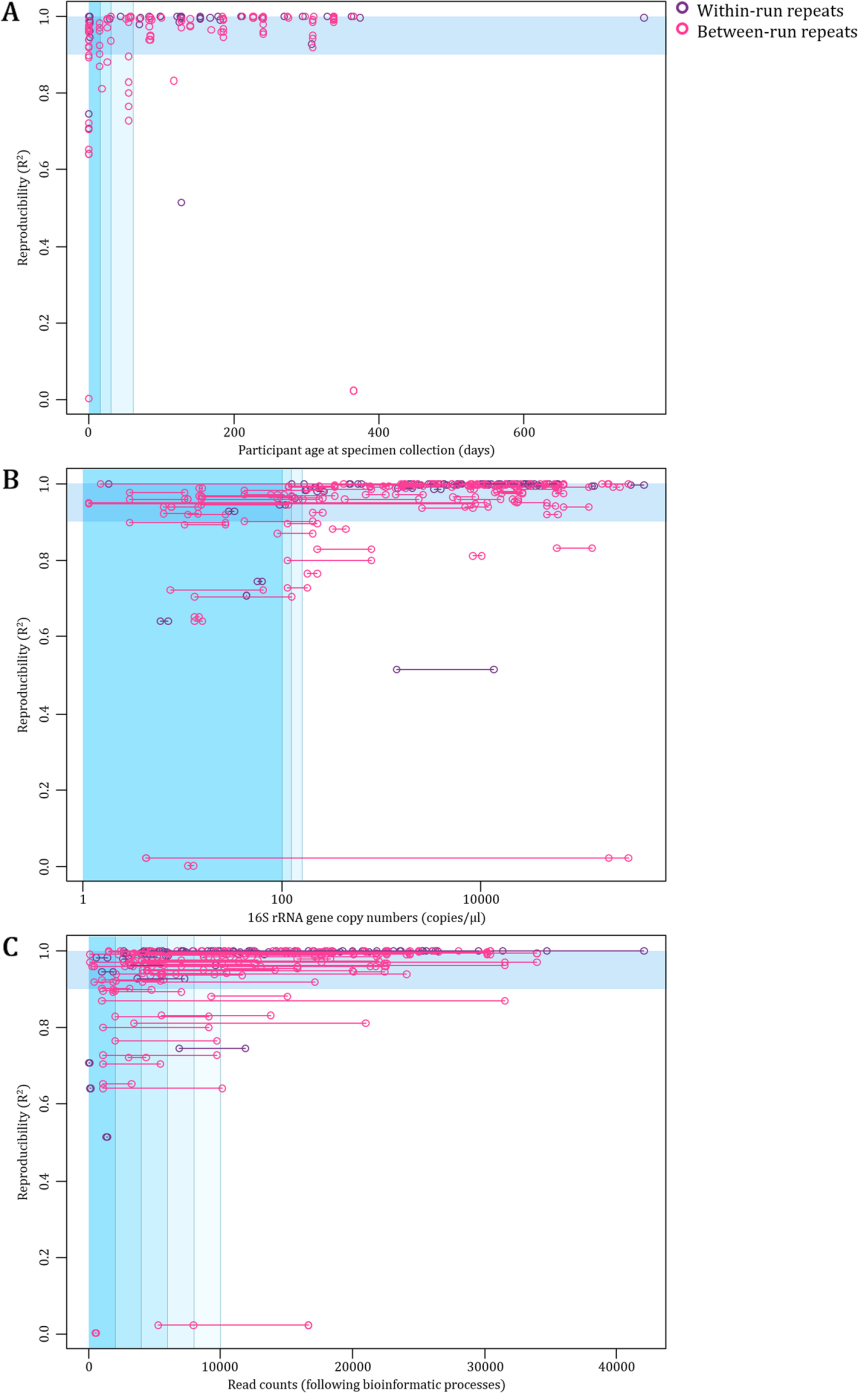
Associations between reproducibility and **a** participant age at specimen collection, **b** 16S rRNA gene copy numbers, and **c** read counts. Reproducibility is measured by coefficient of determination (R^2^) values, calculated by comparing proportions of each OTU present between technical repeats. Horizontal blue bars highlight R^2^ values > 0.90. Different shades of vertical blue bars represent **a**< 7, < 14, < 30, < 60 days; **b**< 100, < 500, < 1000 copies/μl; and **c**< 2000, < 4000, < 6000, < 8000 and < 10,000 reads; respectively. For **b** and **c**, each set of technical repeats had two 16S rRNA gene copy number/read count measures shown as two points connected by a horizontal line on the X-axis

We further investigated whether participant age at specimen collection, 16S rRNA gene copy numbers or read counts could identify sequencing profiles with reduced reproducibility (R^2^ values < 0.90), and hence could be used to identify specimens which should be excluded from analysis. Our results showed associations between participant age at specimen collection (in days), 16S rRNA gene copy numbers (copies/μl) and read counts with R^2^ values (Fig. 7 a-c). Specimens with R^2^ values < 0.90 were collected at a median of 16 days of age (IQR: 0–54), produced 101 16S rRNA gene copies/μl (IQR: 14–355) and 3357 reads (IQR: 1113–9211). In comparison, specimens with R^2^ values > 0.90 were collected at a median of 153 days of age (IQR: 55–240), yielded 5476 16S rRNA gene copies/μl (396–23,231) and 11,891 reads (IQR: 5456–20,045) (Additional file 6).

In summary, low biomass technical repeats - represented by low 16S rRNA gene copy numbers (< 500 copies/μl) - produced fewer reads and were primarily collected during the first 2 weeks of life. These specimens tended to have higher alpha diversity, produce more OTUs (including “spurious OTUs”) and had less reproducible sequencing profiles. In addition, the majority of low biomass technical repeats clustered with Primestore when assessing beta-diversity.

### Improved quality of 16S rRNA gene datasets is attainable via the *decontam* R package for identification of “potential contaminants”

We aimed to validate two in silico approaches (“NTConly” and “NTC + decontam”) to identify potential contaminants from bacterial profiles generated from technical repeats (NP and IS specimens processed in duplicate, triplicate or quadruplicate) (Table 1, Table 2). Prior to analysis, we denoised the dataset consisting of 35 Primestores and 209 technical repeats by removing 249 of the 1252 OTUs identified as “spurious OTUs” across the dataset. We included technical repeats with > 500 16S rRNA gene copies/μl measured from their template (*n* = 148) as true biological specimens (based on results reported in the previous section). The “NTConly” approach designates an OTU as a “potential contaminant” when it is detected across both Primestore (*n* = 35) and technical repeats (*n* = 148) [16, 18, 19, 28]. The “NTC + decontam” approach designates an OTU as a “potential contaminant” via implementation of the *decontam* package in R [29]. We identified potential contaminants via the “NTC + decontam” approach based on 1) their frequency as a function of the specimen biomass; and 2) their prevalence in true biological specimens (*n* = 148) compared to Primestore (*n* = 35).

#### The “NTC + decontam” approach results in less apparent shifts in sequencing profiles of common nasopharyngeal colonizers compared to the “NTConly” approach

We identified 386 OTUs as “potential contaminants” when using the “NTConly” approach (Additional file 7). In comparison, the “NTC + decontam” approach identified 115 OTUs as “potential contaminants”, all of which were also identified using the “NTConly” approach (Additional file 7). We were able to obtain genus-level classifications (Additional file 7; Additional file 8) for 76% (294/386) and 75% (86/115) of OTUs identified from the two approaches, of which the majority [61% (179/294); 66% (57/86)] were previously reported as potential contaminants [15, 30].

A number of OTUs identified as “potential contaminants” using the “NTConly” approach represented species commonly detected in the nasopharynx (Additional file 7; Additional file 8). These included OTU_2 (*Moraxella catarrhalis*), OTU_4 (*Escherichia coli*), OTU_6 (*Staphylococcus aureus*), OTU_7 (*Haemophilus influenzae*), OTU_8 (*Streptococcus pneumoniae*), OTU_10 (*Corynebacterium* spp*.*), OTU_11 (*Pseudomonas aeruginosa*), OTU_14 (*Listeria monocytogenes*), OTU_15 (*Acinetobacter baumannii*) and OTU_29 (*Neisseria lactamica*/*Neisseria meningitidis*). Of these, only OTU_4, OTU_11 and OTU_14 were identified as “potential contaminants” using the “NTC + decontam” approach.

**Fig. 8:**
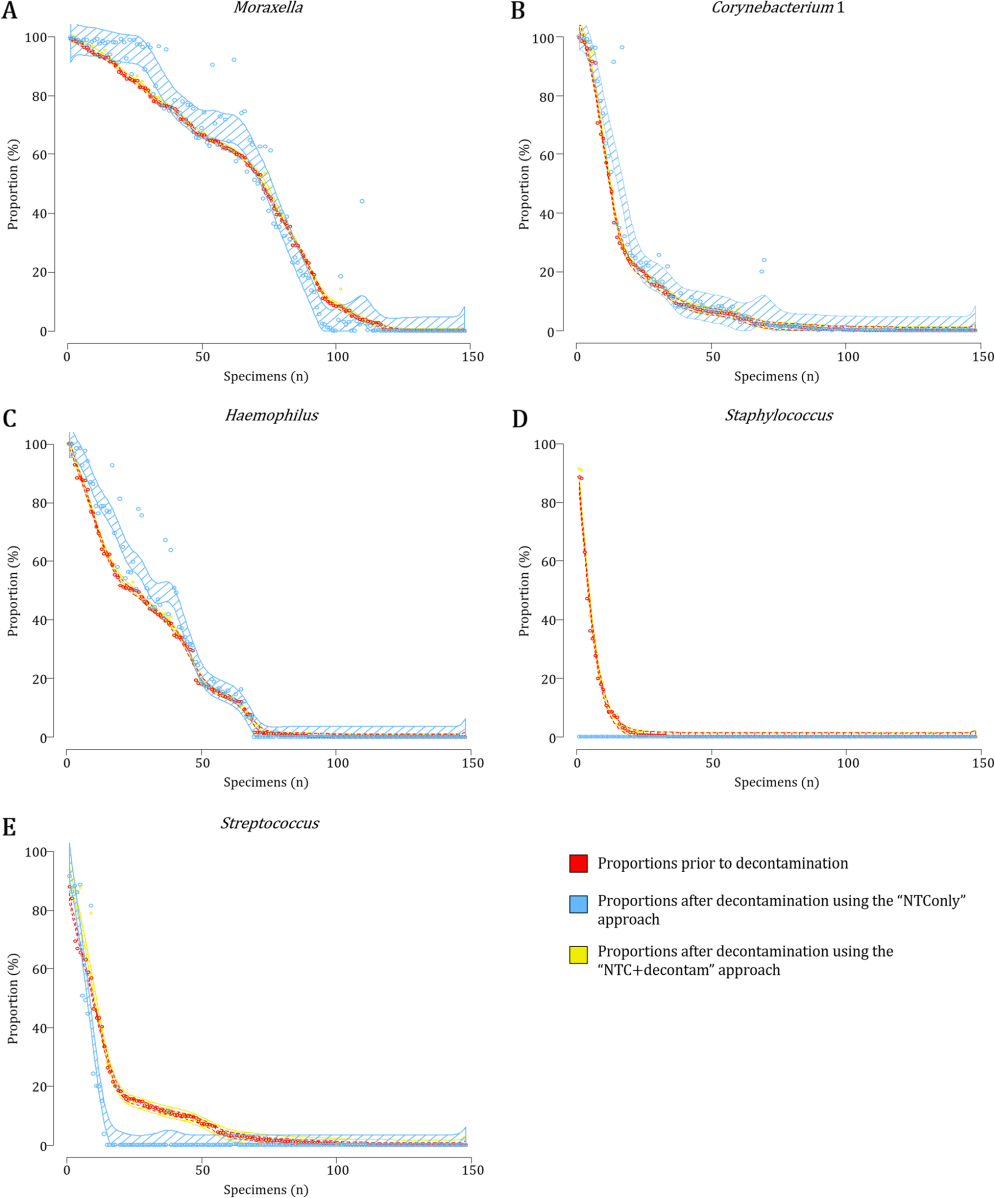
Shifts in profiles of bacterial genera commonly detected from the nasopharynx prior to and following decontamination via two in silico approaches. Per specimen shifts (*n* = 148) in bacterial proportions are shown for bacterial genera commonly detected from the nasopharynx: **a***Moraxella*, **b***Corynebacterium* 1, **c***Haemophilus*, **d***Staphylococcus* and **e***Streptococcus*. Open circles and smoothing splines (representing a factor of 2x the standard deviation) denote bacterial proportions (Y-axis) for each of the specimens (X-axis). Red: Proportions prior to decontamination; Blue: Proportions following the removal of “potential contaminants” identified using the “NTConly” approach; Yellow: Proportions following the removal of “potential contaminants” identified using the “NTC + decontam” approach

Following decontamination steps, we generally observed more dispersed shifts in proportions from bacteria commonly detected from the nasopharynx using the “NTConly” as opposed to the “NTC + decontam” approach (Fig. 8a-e; Additional file 9). For example, we observed complete removal of the *Staphylococcus* genus from 95% of specimens positive (*n* = 104) via the “NTConly” approach compared to 0% when using the “NTC + decontam” approach (Fig. 8 d; Additional file 9). Similarly, the *Streptococcus* genus was removed from 73% of specimens positive (*n* = 143) using the “NTConly” approach compared to 0.7% when using the “NTC + decontam” approach (Fig. 8e; Additional file 9). At OTU-level, the “NTC + Decontam” approach better identified OTU_6 (*S. aureus*), OTU_8 (*S. pneumoniae*) and OTU_1390 (*S. anginosus*) as potential nasopharyngeal colonizers and OTU_23 (*S. equi* subsp. *equi* or *zooepidemicus*) as a potential contaminant (Additional file 10).

#### The “NTC + decontam” approach results in improved removal of “potential contaminants” when compared to the “NTConly” approach

**Fig. 9:**
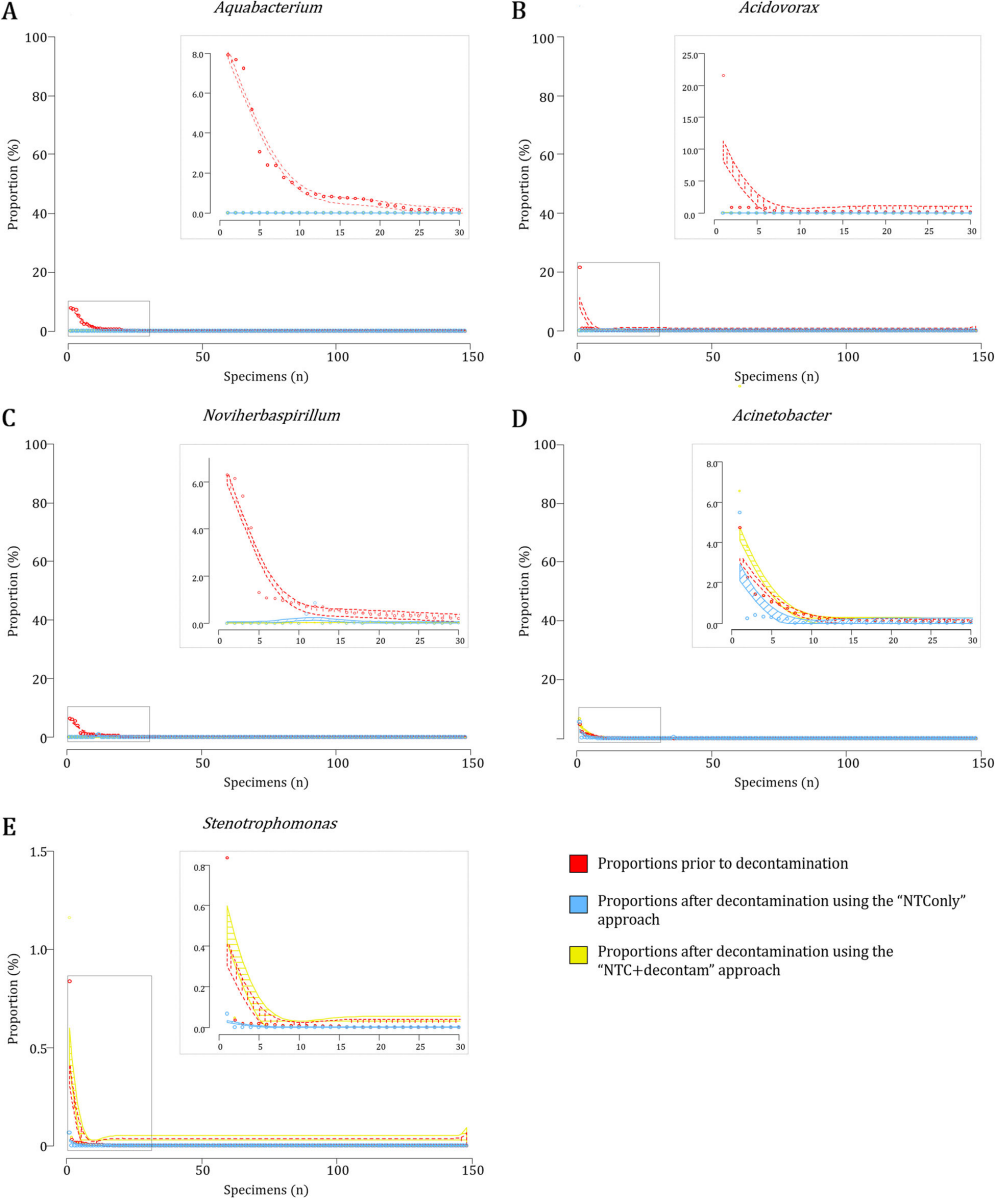
Shifts in profiles of potential contaminants prior to and following decontamination via two in silico approaches. Per specimen shifts (*n* = 148) in bacterial proportions are shown for bacterial genera commonly described as “potential contaminants” in 16S rRNA gene sequencing datasets **a***Aquabacterium*, **b***Acidovorax*, **c***Noviherbaspirillum*, **d***Acinetobacter* and **e***Stenotrophomonas*. Open circles and smoothing splines (representing a factor of 2x the standard deviation) denote bacterial proportions (Y-axis) for each of the specimens (X-axis). Red: Proportions prior to decontamination; Blue: Proportions following the removal of “potential contaminants” identified using the “NTConly” approach; Yellow: Proportions following the removal of “potential contaminants” identified using the “NTC + decontam” approach

Figure 9 summarises the per specimen proportions observed for five genera previously reported as “potential contaminants” prior to and after decontamination (Additional file 9). Both “NTConly” and “NTC + decontam” approaches completely removed *Aquabacterium*, *Acidovorax* and *Noviherbaspirillum* profiles from the majority (100, 99 and 72%) of specimens positive for these genera (Fig. 9a-c; Additional file 9). We observed lower proportions for specimens positive for *Acinetobacter* and *Stenotrophomonas* following decontamination using the “NTConly” compared to the “NTC + decontam” approach (Fig. 9d-e; Additional file 9). Both *Acinetobacter* and *Stenotrophomonas* have been reported as potential contaminants at genus-level in 16S rRNA gene sequencing datasets [15, 30], but are also reported as of the upper airway bacteria [31–33]. When investigating both *Acinetobacter* and *Stenotrophomonas* genera at OTU-level, we observed that the “NTConly” approach identified OTU_15 (*A. baumannii*), OTU_863 (*A. johnsonii*), OTU_358 (*A. calcoaceticus-A. baumannii* complex) and OTU_56 (*S. maltophilia*) (Additional file 10) as potential contaminants, all of which are commonly isolated from the respiratory tract.

In summary, the “NTConly” approach to decontamination resulted in larger shifts in proportions of genera commonly detected from the nasopharynx compared to using the *decontam* package implemented through R software (“NTC + decontam” approach). Furthermore, the “NTC + decontam” approach better differentiated likely “true nasopharyngeal bacteria” from “potential contaminants” at OTU-level compared to the “NTConly” approach.

## Discussion

The complexities of 16S rRNA gene sequencing of low biomass specimens are increasingly recognised and have broad applicability [13, 14, 30, 34]. In order to deal with such complexities, we provide, in a stepwise manner, a comprehensive overview of several key components of a quality control process for low biomass 16S rRNA gene sequencing studies. We highlight the importance of evaluating DNA extraction protocols prior to the implementation thereof, and the need to account for potential effects of different storage buffers used to preserve specimens. We further show the value of determining specimen exclusion criteria via the use of optimal sequencing controls and exploratory analyses and highlight the importance of optimized approaches for contaminant identification and removal in any low biomass 16S rRNA gene sequencing study. We believe that our specific and detailed approach to dealing with low biomass respiratory tract specimens provides a rigorous method to assessing and responding to the complexities inherent to low biomass 16S rRNA gene sequencing experiments in general.

Prior to the advent of 16S rRNA gene high-throughput sequencing, bacterial DNA extraction platforms have primarily been evaluated based on DNA yield and purity, DNA shearing, bacterial diversity (using fingerprinting techniques) and how well DNA from hard- and easy-to-lyse bacterial cells are represented (using targeted PCR). Recently, mock community controls have been implemented to further assess the performance of extraction platforms [35, 36]. When carefully selected, these mock controls may allow for the evaluation of extraction efficiency, serve to determine extraction reproducibility, and provide insight into contamination profiles. However, in order evaluate these important aspects of 16S rRNA gene sequencing, mock community controls need to represent bacterial communities expected from the specimen of interest and optimally reflect its biomass. In this study, we used both high and low biomass bacterial mock community controls to evaluate the performance of two DNA extraction kits. Overall, specimen biomass had a noticeable impact on sequencing profiles irrespective of the storage buffer and DNA extraction method used. These findings, together with previous reports on breast milk and faecal 16S rRNA gene sequencing profiles [37, 38], emphasise the importance of careful consideration of the type of specimen and its biomass when performing these comparisons. For example, previous studies have shown that low biomass breast milk specimens are influenced by contaminants and the DNA extraction method used [37], whilst diluted faecal specimen microbiota profiles are not much affected by contamination but largely impacted by the DNA extraction method used [38]. Our study showed that specimen biomass is a primary driver of sequencing profiles, and that the effect of DNA extraction kits become more evident with reduced specimen biomass. We further evaluated the effect of different storage buffers on sequencing profiles as storage buffers from different manufacturers may contain different levels of endogenous contaminants (contaminants introduced during the manufacturing process). Again, the effect of storage buffers on bacterial mock community profiles was more apparent among low biomass controls, which further highlights the importance of careful consideration when selecting NTCs for these types of studies [30]. If selected carefully, NTCs will ameliorate the detection and removal of “background OTUs”, whether inherent to storage buffers, or originating from cross-contamination between wells [20] or DNA extraction methods used [37, 38].

In addition to evaluating DNA extraction kits and storage buffers, 16S high-throughput studies also need to consider potential “batch effects” between independent runs. Large-scale experiments processed over long periods of time are highly susceptible to batch effects introduced by reagents (DNA extraction kits, PCR reagents and sequencing kits), laboratory personnel and laboratory environment [18, 39]. Although downstream statistical tools have been introduced to correct for batch effects [40], it is crucial to minimize the probability of confounding during wet lab processes. Sources of variation arising from different biological groups (for example, specimens collected at different ages, from cases and controls, or from intervention and control groups) should be distributed evenly across different batches to avoid batch-effect biases. In order to evaluate batch effects across different runs, researchers need to carefully consider sequencing controls most suitable for their study design. Studies have shown that bacterial mock community controls are not only useful for evaluation of DNA extraction processes, amplification and sequencing steps [41–44], but may also provide insight into reproducibility across runs [45]. Although we support the use of mock community controls for reproducibility assessment [30]; these controls may not accurately represent inherent features of the biological specimens under study and should be supplemented with replicate biological specimens. Our study provides comparisons between biological specimens repeated within and between different sequencing runs and highlight their role in improving the quality of 16S rRNA gene sequencing data generated from low microbial biomass specimens [30]. As expected, our study showed slightly higher reproducibility between specimens repeated within the same runs compared to specimens repeated across different runs. However, we found that the biological nature of NP and IS specimens under investigation had a larger effect on reproducibility. Biological specimens of low biomass (< 500 16S rRNA gene copies/μl) showed lower sequencing reproducibility [46]. This also correlated with the age at which specimens were collected - the majority of specimens with low reproducibility (and low biomass) were collected during the first 2 weeks of life. The latter is an important observation as analyses may be biased when including “early life” specimens without incorporating the types of quality checks we report here. Hence, the sole use of bacterial mock communities may not be sufficient to determine reproducibility of low biomass specimens. Of note, correlations between bacterial biomass and reproducibility measures are only reliable when using appropriate quantification tools and we highly recommend the use of qPCR approaches targeting the 16S rRNA gene [47]. In support of the latter, studies have shown a general over-estimation of DNA concentrations measured by the NanoDrop® ND-1000 Spectrophotometer in comparison to fluorescence-based quantification methods such as the Qubit [48, 49], whilst fluorescence-based quantification methods are less accurate than qPCR approaches – particularly for low biomass specimens [50–52].

Low biomass biological specimens produce less reproducible sequencing profiles compared to high biomass specimens as a result of contaminant profiles originating from reagents or the laboratory environment [15–18], and from neighbouring high biomass specimens [20]. Cross-contamination between wells may be minimised by the use of individual tubes as opposed to plates during DNA extraction and amplification processes, whilst reagent contaminants introduced during amplification processes may be reduced by PCR master mix decontamination protocols [53, 54]. However, these precautionary steps may still need to be supplemented with in silico identification and removal of contaminants when dealing with low biomass specimens. In order to optimally identify and apply in silico decontamination to sequencing datasets, NTCs need to be selected carefully [16, 55]. As endogenous NTC contaminant profiles are introduced to very low biomass biological specimens during the process of specimen collection and storage, neat storage buffers should be processed alongside biological specimens. In the event that storage buffers are not used to preserve specimens, the DNA extraction kit lysis buffer could be implemented as NTC during the process of DNA extraction. If DNA extraction kit lysis buffer is used as NTCs, it is recommended that the lysis buffer is exposed to items used during specimen collection (such as collection tubes, swabs, scoops, etc.) prior to the process of DNA extraction. The use of water as NTCs during DNA extraction steps is not recommended, as it may introduce external contaminant profiles which are not representative of the specimens or the DNA extraction kit [56–58]. Both exogenous and endogenous contaminant profiles present in NTCs add to the complexity of in silico contaminant identification as they may differ across batches of specimens collected, batches of reagents ordered, batches of DNA extractions and batches of library preparation. This highlights the importance of including a good representative of NTCs with each sequencing project - starting with specimen collection and ending with downstream in silico analyses.

Our study further showed that the in silico approach taken to identify and remove potential contaminants is just as important as identifying and including a good representative of NTCs during specimen collection, DNA extraction, library preparation and sequencing steps. We compared decontamination results from a widely used method in which all OTUs present in NTCs are flagged as contaminants and removed from biological specimens [16, 18, 19, 28] (“NTConly” approach) to a more sophisticated method using the *decontam* package [29] (“NTC + *decontam”* approach). To date, few studies have performed these types of comparisons, especially in relation to respiratory tract specimens. In addition, we evaluated these two in silico decontamination processes by subtracting the maximum proportion of each OTU identified as a contaminant among NTCs, as opposed to the complete removal thereof. The *decontam* package [29] provides a statistical classification method for identifying contaminants derived from biological profiles based on their DNA concentrations [17, 18, 21, 35, 59]. This approach appeared to perform well on our dataset which showed clear trends between specimen biomass, diversity, reproducibility and the frequency of spurious OTUs. Our results showed that the identification of potential contaminants from NTCs without the *decontam* package (“NTConly” approach) more frequently identified bacteria commonly isolated from the nasopharynx as potential contaminants [15, 60, 61]. In contrast, the *decontam* package allowed for better discrimination between likely “true” and “contaminant” profiles at OTU-level, e.g., OTUs representing *S. equi* [62] but not *S. pneumoniae*, were identified by the “NTC + *decontam”* approach. This emphasises the importance of investigating sequencing data below genus-level resolution [using OTUs or amplicon sequence variants (ASVs)] in addition to genus-level analysis when considering potential contaminants. The result obtained for OTU_56 (*S. maltophilia*) and others, for example OTU-358 (*A. calcoaceticus-A. baumannii* complex), OTU-1390 (*S. anginosus* group) and OTU_863 (*A. johnsonii*), further highlight potential risks of using a relative abundance threshold to remove low abundant reads in silico [35]. All above-mentioned OTUs had less than 100 reads sequenced per specimen, however, may represent rare features truly present in biological specimens [31–33, 63]. Also, by randomly setting thresholds to remove only low abundant reads from the dataset will not remove abundant contaminants which poses higher risks for downstream analysis interference. Finally, our results do not support the use of a “blacklist” approach to remove reads or taxa previously reported as common contaminants [21, 22]. For example, at genus-level, *Streptococcus*, *Staphylococcus*, *Haemophilus*, *Corynebacterium*, *Acinetobacter* and *Stenotrophomonas* have all been listed as potential contaminants by previous reports [15, 30], but are also common indigenous bacteria of the nasopharynx.

There are several limitations to this study. In the first section of our paper, only two DNA extraction kits were evaluated which do not represent the current assortment of kits available for DNA extraction from biological specimens. In addition, these kits were not selected for evaluation based on their aptness to extract DNA from low biomass specimens, but rather based on the fact that they were readily available for use in our laboratory. Further, limited numbers of repeats were included as mock controls during the evaluation of extraction methods. In the second section of our paper, we primarily included NP specimens to validate sequencing performance with limited numbers of IS specimens included for repeat processing. Finally, in the last section of our paper, we only assessed two in silico approaches for the identification of “potential contaminants”. We did not investigate the use of tubes as opposed to plates during DNA extraction and amplification steps, nor did we investigate PCR master mix decontamination protocols [53, 54] prior to amplification and sequencing.

## Conclusions

We have described a stepwise approach to ensuring reliable, reproducible results from 16S rRNA gene sequencing of low biomass respiratory specimens. In our approach, we 1) investigate the performance of DNA extraction kits and the use of different storage buffers on low and high biomass mock community controls; 2) include relevant NTCs representing potential background OTUs present in biological specimens that undergo the same processing steps as biological specimens; 3) account for batch effects by including adequate sequencing controls; 4) establish specimen exclusion criteria based on reproducibility measures and clustering patterns as functions of specimen biomass, demographic data (such as “participant age at specimen collection”), and/or read counts; 5) denoise sequencing data by removing spurious OTUs; and 6) explore different in silico approaches to best correct for contamination. We show the value of detailed exploratory analyses of sequencing controls to identify and reduce experimental error.

## Methods

We processed NP and IS specimens included in this study over a total of eleven sequencing runs as part of a study nested within the Drakenstein Child Health Study (DCHS) [26]. The DCHS is a population-based birth cohort study in a peri-urban area of South Africa which investigates the role of low biomass NP and IS microbial communities in the development of lower respiratory tract infection or wheezing illness during infancy and childhood. Different types of analyses, extraction and sequencing controls were included to address the three objectives of this study. A reference guide to DNA extraction kits, storage buffers/no template controls, bacterial mock communities, technical repeats and decontamination approaches used in this study is provided in Table 1.

### Extraction and sequencing controls

In summary, each of the eleven sequencing runs processed as part of the study nested within the DCHS [26] consisted of four 96-well plates. We included controls for DNA extraction (“extraction controls”) (Tables 1 and 2) and other downstream processes including PCR amplification, library preparation and sequencing steps (“sequencing controls”) (Tables 1 and 2).

#### Extraction controls

We addressed the first objective of our study by including four bacterial mock communities as extraction controls across two of the eleven sequencing runs (Table 2). We used ZymoBIOMICS™ Microbial Community Standard bacterial cells (Catalog No. D6300, Zymo Research Corp., Irvine, CA, United States) (“Zymobiomics-Cells”) to generate the four bacterial mock communities (Table 2, Additional file 1). According to the manufacturer’s specifications, Zymobiomics-Cells consist of three gram-negative bacteria within the phylum Proteobacteria [*Pseudomonas aeruginosa* (theoretical composition in terms of 16S rRNA gene abundance: 4.6%), *Escherichia coli* (10%) and *Salmonella enterica* (11.3%)] and five gram-positive bacteria within the phylum Firmicutes [*Lactobacillus fermentum* (18.8%), *Enterococcus faecalis* (10.4%), *Staphylococcus aureus* (13.3%), *Listeria monocytogenes* (15.9%) and *Bacillus subtilis* (15.7%)]. Zymobiomics-Cells were mixed with two storage buffers [PrimeStore® Molecular Transport medium (“Primestore”) [64, 65] and a medium containing skim milk, tryptone, glucose, and glycerine (“STGG”)], to further evaluate the effect of different storage buffers on 16S rRNA gene sequencing profiles generated from two DNA extraction methods. Primestore is used as a storage buffer for NP and IS specimens collected for molecular testing by the parent DCHS study [65], whilst STGG is widely used to preserve NP specimens intended for culture [65, 66] and has also been used for 16S rRNA gene sequencing [67]. In order to limit reagent batch effects, we respectively pooled Primestore and STGG buffers with corresponding batch numbers prior to their use.

We generated two bacterial mock communities by suspending 900 μl of Zymobiomics-Cells in 3600 μl of each of the storage buffers, respectively (Additional file 1). These bacterial mock communities represented high biomass bacterial communities (“Zymobiomics-Primestore-high” and “Zymobiomics-STGG-high”) (Table 2, Additional file 1). We further made a 1-in-10^4^ fold dilution of each of the two high biomass bacterial mock communities (Table 2, Additional file 1), each representing low biomass bacterial communities (“Zymobiomics-Primestore-low” and “Zymobiomics-STGG-low”). High and low biomass bacterial mock communities were included to evaluate the effect of bacterial biomass on resultant bacterial profiles following the use of two DNA extraction methods (Table 2, Additional file 1).

Using two extraction methods (automated and manual), we extracted DNA in triplicate from each of the four bacterial mock communities (Zymobiomics-Primestore-high, Zymobiomics-STGG-high, Zymobiomics-Primestore-low and Zymobiomics-STGG-low). In preparation for DNA extraction using the automated method, we vortexed the bacterial mock communities for 5 s and transferred 400 μl of homogenised bacterial mock communities to ZR BashingBead™ Lysis Tubes containing 0.5 mm bashing beads (catalogue no. ZR S6002–50, Zymo Research Corp., Irvine, CA, United States). We performed a mechanical off-board lysis step at 50 Hz for 5 min using the TissueLyser LT™ (Qiagen, FRITSCH GmbH, Idar-Oberstein, Germany). We centrifuged the lysate at 10000 rpm for 1 min and loaded 250 μl of the supernatant onto the QIAsymphony® SP instrument (Qiagen, Hombrechtikon, Switzerland) for DNA extraction. We used the DSP Virus/Pathogen Mini Kit® (catalogue no. 937036, Qiagen GmbH, Hilden, Germany) (“Kit-QS”) to carry out automated extractions of DNA with the elution volume set to 60 μl. For manual DNA extractions, we followed the manufacturer’s instructions as outlined by the ZymoBIOMICS DNA Miniprep Kit (catalogue no. ZR D4300, Zymo Research Corp., Irvine, CA, United States) (“Kit-ZB”). We vortexed the bacterial mock communities for 5 s and transferred 250 μl of homogenised bacterial mock communities to ZR BashingBead™ Lysis Tubes containing a mix of 0.1 and 0.5 mm bashing beads (catalogue no. ZR S6012–50, Zymo Research Corp., Irvine, CA, United States) together with 750 μl ZymoBIOMICS™ Lysis Solution as per manufacturer’s instructions. We performed a mechanical lysis step at 50 Hz for 5 min using the TissueLyser LT™ (Qiagen, FRITSCH GmbH, Idar-Oberstein, Germany) before continuing with the manual extraction steps as outlined by the manufacturer’s protocol. We eluted the DNA in a final volume of 100 μl as per manufacturer’s recommendations. We processed neat Primestore and STGG as no template extraction controls (Table 2) alongside the four bacterial mock communities using the two methods described above. All NTC extraction aliquots were obtained from the respectively pooled Primestore and STGG buffers used to generate the bacterial mock communities. This was done to limit reagent batch effects as the primary aim was to determine the effect of different storage buffers and bacterial biomass on extraction profiles using two DNA extraction kits.

#### Sequencing controls

We allocated six wells per 96-well plate to sequencing controls during each of the eleven sequencing runs. Sequencing controls included “bacterial mock community DNA”, “technical repeats” and “no template controls” (“NTCs”) (Table 2). Technical repeats and NTCs were used to address the second and third objectives of our study.

Bacterial mock community DNA are commercially available pre-extracted genomic DNA mixtures from bacterial communities which did not undergo DNA extraction in our laboratory. We included a minimum of one of two types of bacterial mock community DNA controls per 96-well plate across the eleven runs: 1) 1-in-10 fold dilutions of HM-783D (BEI Resources, NIAID, NIH as part of the Human Microbiome Project, Manassas, VA, USA) (“BEI-DNA”) and 2) 1-in-10 fold dilutions of ZymoBIOMICS™ Microbial Community DNA Standard (catalogue no. D6305, Zymo Research Corp., Irvine, CA, United States) (“Zymobiomics-DNA”) (Table 2). HM-783D represents a staggered mixture of genomic DNA from 17 genera (theoretical 16S rRNA gene composition: *Pseudomonas aeruginosa* (2.2%), *Escherichia coli* (21.9%), *Rhodobacter sphaeroides* (21.9%), *Clostridium beijerinckii* (2.2%), *Streptococcus agalactia, S. mutans and S. pneumoniae* (24.1%), *Staphylococcus aureus and S. epidermidis* (24.1%), *Bacillus cereus* (2.2%), *Acinetobacter baumannii* (0.2%), *Neisseria meningitidis* (0.2%), *Lactobacillus gasseri* (0.2%), *Listeria monocytogenes* (0.2%), *Helicobacter pylori* (0.2%), *Propionibacterium acnes* (0.2%), *Enterococcus faecalis* (0.02%), *Bacteroides vulgatus* (0.02%), *Actinomyces odontolyticus* (0.02%) and *Deinococcus radiodurans* (0.02%) [27]). ZymoBIOMICS™ Microbial Community DNA Standard represents an even mixture of genomic DNA from eight genera (theoretical 16S rRNA gene composition: previously described for Zymobiomics-Cells in the DNA extraction section above). For the purpose of this study, we only report on data from BEI-DNA (*n* = 3) and Zymobiomics-DNA (*n* = 8) processed in the same sequencing runs as the bacterial mock communities used to compare DNA extraction methods. Zymobiomics-DNA was used as a reference when evaluating two DNA extraction methods.

Technical repeats refer to DNA extracts from NP and IS specimens, randomly selected for repeat amplification and sequencing across the eleven runs (Table 2). We extracted DNA from all NP and IS specimens processed across the eleven runs using the automated extraction platform and Kit-QS, as described above. We randomly selected a single biological specimen’s DNA extract from any of the 90 biological specimens processed per 96-well plate for repeat amplification and sequencing (“within-run repeats”). We further randomly selected a single biological specimen’s DNA extract from any of the 360 biological specimens processed per run for repeat amplification and sequencing on an independent sequencing run (“between-run repeats”). Technical repeats were used to validate 16S rRNA gene sequencing data from low biomass specimens and to investigate decontamination processes.

In addition to technical repeats, we selected neat Primestore extracted using Kit-QS (processed alongside NP and IS specimens) as NTCs. The latter was included as NTCs due to the fact that NP and IS specimens collected for molecular analyses in the DCHS parent study (also included as technical repeats in this study) are stored in Primestore and extracted using Kit-QS [65]. We processed a minimum of one Primestore per 96-well plate, but only included Primestores with 16S rRNA gene quantification data for downstream analysis (Table 2). All Primestores included in this study underwent DNA extraction, PCR amplification, library preparation and sequencing steps alongside technical repeats. Primestores processed across the eleven sequencing runs were used to evaluate the quality of 16S rRNA gene sequencing data and to correct for potential contamination inherent to 16S rRNA gene sequencing. For the purpose of comparing different extraction methods, we also included Primestore and STGG extracts (each extracted in triplicate using Kit-QS and Kit-ZB) processed alongside extraction controls (Table 2).

### Amplicon library preparation and Illumina sequencing

We measured DNA yield and purity from extraction and sequencing controls using the NanoDrop® ND-1000. We further determined total bacterial load present in extraction and sequencing controls using a previously described qPCR method targeting the 16S rRNA gene [47].

We amplified the V4 hypervariable region of the 16S rRNA gene using a two-step amplification approach [68]. A total of 7 μl of DNA from all extraction and sequencing controls was included as template during the first PCR. During the second PCR, we used 7 μl of the PCR product from the first PCR as template to add adapters, barcodes, 12–15 staggered nucleotides and priming regions [68]. PCR conditions and modified primers used in the two-step amplification approach have previously been published [68, 69].

We purified amplicons by adding Agencourt® AMPure® XP PCR Purification solution (catalogue no. A63881, Beckman Coulter, CA, USA) to amplicons from the second PCR at a 0.65:1 (bead:amplicon) ratio [68]. We used agarose gel electrophoresis and the GloMax®-Multi Detection System (Promega Corporation, Madison, WI, USA) together with the QuantiFluor® dsDNA System (catalogue no. E2670, Promega Corporation, Madison, WI, USA) to verify and quantify amplicons. Following quantification of amplicons, we pooled amplicons from each run (384 wells per run) at 70 ng and purified the pool using Agencourt® AMPure® XP PCR Purification solution at a 1:1 ratio. We quantified the purified pool using the Qubit® Fluorometer (Invitrogen, Life Technologies, CA, USA) and Qubit™ dsDNA BR Assay Kit (catalogue no. Q32850, Invitrogen, Life Technologies, CA, USA). We loaded a total of 7000 ng of the purified pooled library on a 1.6% agarose gel and performed electrophoresis at 35 Voltz for 30 min, 40 Voltz for 45 min, 70 Volts for 180 min and 50 Voltz for 60 min. Purification of the excised 16S library using the QIAquick Gel Extraction kit (QIAgen, MA, USA) followed agarose gel electrophoresis [68].

For each of the eleven runs, we quantified libraries and determined their fragment sizes using the KAPA Library Quantification Kit (catalogue no. KK4844, KAPA Biosystems, Boston, MA, USA) and the Agilent DNA 1000 kit (Agilent Technologies, CA, USA), respectively. We diluted libraries to 4 nM using Buffer EB (Qiagen, Hilden, Germany), after which we denatured and neutralized libraries using 0.2 N NaOH and HT1 hybridization buffer. Over the span of eleven sequencing runs, we gradually increased library concentrations with each run (ranging between 4 pM and 7 pM per library) to reach optimal flow cell loading concentrations. Each sequencing run contained the PhiX internal sequencing control spiked at 15%. We loaded denatured libraries onto the MiSeq Reagent Kit v3 (600-cycle) (Illumina, San Diego, CA, USA) and performed sequencing on the Illumina® MiSeq™ platform as per manufacturer’s instructions [70, 71].

### Bioinformatic steps

We used FastQC and MulitQC packages [72, 73] to assess sequence quality of FASTQ files. We merged paired-end sequence reads and performed quality filtering using the UPARSE algorithm in USEARCH version 10.0; whereby UPARSE merge_fastq (fastq_maxdiff set to 3) and UPARSE filter_fastq (sequences truncated to 250 bp and fastq_maxee set to 0.1) commands were used, respectively [74]. We used USEARCH10 sortbysize [75] to de-replicate sequences occurring more than once; USEARCH10 cluster_otus (clustering radius set to 3) [75] to cluster sequences into operational taxonomic units (OTUs), USEARCH10 uchime2_ref tool [75] to remove chimeras; and USEARCH10 usearch-global [75] to determine OTU counts. We used the USEARCH10 uchime2_ref tool for further chimera detection and removal in addition to the USEARCH10 cluster_otus command as it provides a reference for common chimeric sequences, considering the low biomass nature of specimens included in our study. We used the Quantitative Insights Into Microbial Ecology (QIIME 1.9.0) suite of software [76] to assign taxonomy (using SILVA database [77, 78] and a sequence similarity set to 97%) via the RDP classifier method [79] and the assing_taxonomy.py command [76]. The Nextflow tool [80] was used to loop the bioinformatics processing workflow.

### Data analysis

We used R software version 3.5.1 and RStudio software version 1.1.456 [81] for data analysis and visualisation. Prior to analyses, we transformed count data to compositional data [82–84]. We conducted all analyses at OTU-level.

In order to evaluate the effect of two DNA extraction methods (Kit-QS and Kit-ZB) on 16S rRNA gene bacterial profiles, we compared sequencing profiles from DNA extracts from high and low biomass bacterial mock communities and no template controls (NTCs). Each of these sets of controls were generated using two storage buffers (Primestore and STGG). We used Principal Coordinate Analysis of beta diversities such that the distances between points approximate the beta diversity between each pair. We conducted Permutational Multivariate Analysis of Variance (PERMANOVA) using the function adonis from the package *vegan* [85] on beta diversities with 1000 permutations. We further evaluated the performance of the two DNA extraction methods by comparing how efficiently they extract hard- and-easy-to-lyse bacteria in relation to the reference profile (Zymobiomics-DNA). Since the data is compositional, the isometric logratio transformation (ilr) was applied with the [pivotCoord] function in the R package *robCompositions* [86] such that the bacteria of interest represented the pivot coordinate. This result in the data being represented in an equivalent Euclidean space where single factor analysis of variance (ANOVA) is performed, and Tukey Honest Significant Difference simultaneous confidence intervals computed.

We evaluated the quality of 16S rRNA gene sequencing data from low biomass specimens by comparing OTU-level profiles [including alpha diversity (Shannon diversity index [87])] generated from technical repeats and Primestore]. We investigated correlations between specimen biomass and specimen features including participant age at specimen collection, read counts and alpha diversity. Lambda scaled [88] logarithm of ratio-transformed data (log-ratio) biplots (incorporating data adjusted in a Bayesian context to remove zeros [89–91]), were used to compare bacterial profiles obtained from technical repeats and Primestore. The [vegdist] function offered by the *vegan* package [85] in R was used to calculate the Bray Curtis dissimilarity index [92–95] required for complete linkage (furthest neighbour) clustering analyses. The [hclust] function offered by die *stats* package [81] in R was used to perform unsupervised agglomerative clustering analysis at OTU-level. We calculated sequencing reproducibility [coefficient of determination in linear regression analysis (R^2^)] by comparing the proportions of each OTU present in a specimen to proportions present in their technical repeats [96].

We addressed two in silico approaches to correct for potential contamination inherent to 16S rRNA gene sequencing using technical repeats and Primestore in three steps. The first step entailed denoising of the dataset. We removed “spurious OTUs” (defined as OTUs with < 5 reads across all sequenced technical repeats and Primestore) from the dataset. During the second step, we removed biological specimens with 16S rRNA gene copy numbers < 500/μl as these low biomass specimens produced sequencing profiles with poor reproducibility. The 16S rRNA gene copy number cut-off of < 500/μl was based on data generated by this study (summarised in the previous section), which corresponds with findings from previous reports [46]. Following steps 1 and 2, we compared two in silico approaches (“NTConly” and “NTC + Decontam”) for identifying “potential contaminants” from the dataset generated. Using the “NTConly” approach, we compared all OTUs sequenced from Primestore to those sequenced from technical repeats. If we observed a match between OTUs present in both Primestore and technical repeats, we referred to these OTUs as “potential contaminants”. We subtracted the maximum proportions of each contaminant OTU present in Primestore from technical repeats. The second approach, “NTC + Decontam”, used the *decontam package* in R [29] to identify potential contaminants. We implemented the isContaminant function [29] and a combination of the “frequency- and prevalence-based methods” offered by the *decontam* package [29]. The “frequency-based method” identifies contaminants based on the frequency of each OTU as a function of the concentration of specimen biomass. The “prevalence-based method” offered by the *decontam* package identifies contaminants based on the prevalence of each OTU in true positive (biological) specimens versus the prevalence in NTCs [29]. Following the identification of potential contaminants using the *decontam* package, we subtracted the maximum proportions of each contaminant OTU present in Primestore from technical repeats.

## Supplementary information

**Additional file 1.** Four bacterial mock communities used to evaluate the effect of DNA extraction methods, storage buffers and bacterial biomass on 16S rRNA gene sequencing profiles. Zymobiomics-Primestore-high and Zymobiomics-Primestore-low: ZymoBIOMICS™ Microbial Community Standard bacterial cells in DNA/RNA Shield™ (Zymobiomics-Cells) suspended in PrimeStore® Molecular Transport medium (Primestore); Zymobiomics-STGG-high and Zymobiomics-STGG-low: Zymobiomics-Cells suspended in skim-milk tryptone glucose glycerol transport medium (STGG).

**Additional file 2. **Operational taxonomic units (OTUs) sequenced from bacterial mock community DNA controls. Manufacturers’ specified versus observed OTU composition from BEI-DNA (A-B) and Zymobiomics-DNA (*n* = 8) (C-D) mock controls. Panels A and C represent OTUs from bacterial genera detected at mean proportions of > 0.5% from BEI-DNA (A) and Zymobiomics-DNA (C), respectively. Panels B and D represent OTUs from bacterial genera detected at mean proportions of < 0.5% from BEI-DNA (B) and Zymobiomics-DNA (D), respectively. Bacterial genera in grey font are expected in the bacterial mock community DNA controls but missing from the profiles generated in our laboratory. Bacterial genera in red font are not expected in mock community DNA. OTUs in red font are not expected in mock community DNA or unclassifiable at species-level. Bacterial genera are colour-coded according to the phylum to which they belong (Shades of red: *Firmicutes*; shades of blue: *Proteobacteria*; shades of yellow: *Actinobacteria*; olivegreen: *Bacteroidetes*; seagreen: *Cyanobacteria*; purple: *Deinococcus-Thermus* and grey: unclassified).

**Additional file 3. **Bacterial composition of Zymobiomics-DNA (n = 8) compared to Zymobiomics-Primestore-high (*n* = 6) and Zymobiomics-STGG-high (n = 6). The two high biomass mock communities, Zymobiomics-Primestore-high and Zymobiomics-STGG-high, represent triplicate extractions using two extraction methods (blue filled circles: Kit-QS and red filled circles: Kit-ZB). Zymobiomics-DNA (darkgreen filled circles) were included to validate sequencing profiles generated using the two extraction methods. Unsupervised hierarchical clustering distances are based on Bray Curtis dissimilarity indices calculated at OTU-level. Differences between bacterial mock controls are shown at genus-level, with colour-codes representing phylum-level classification (Shades of blue: *Proteobacteria*, shades of red: *Firmicutes*). Genera with proportions < 1% in each of the specimens are grouped together as “Other” and shown in grey.

**Additional file 4.** Differences between hard-and-easy to lyse bacterial profiles from Zymobiomics-DNA (n = 8) and extracts from high bacterial mock community controls [Zymobiomics-Primestore-high (n = 6) and Zymobiomics-STGG-high (n = 6)] using Kit-QS and Kit-ZB. A) The Tukey Honest Significant Difference simultaneous confidence intervals calculated at OTU-level indicate whether bacterial profiles extracted from high biomass bacterial mock community controls using Kit-QS and Kit-ZB differ significantly from Zymobiomics-DNA, and B) between Kit-QS and Kit-ZB. Confidence intervals computed on the isometric logratio transformation (ilr) scale indicates statistical significance at a 5% significance level when it excludes zero. Blue confidence intervals: significant findings for Kit-QS; Red confidence intervals: significant findings for Kit-ZB.

**Additional file 5. **Sequencing output from technical repeats (*n* = 209) stratified by 16S rRNA gene copy numbers and participant age at specimen collection.

**Additional file 6.** Sequencing reproducibility is associated with participant age at specimen collection, 16S rRNA gene copy numbers and read counts.

**Additional file 7.** Genus-level classification of OTUs identified as potential contaminants using “NTConly” and “NTC + decontam” in silico approaches.

**Additional file 8.** Summary of FASTA sequences for each OTU classified in the dataset.

**Additional file 9.** OTU- and genus-level proportions prior to (“No decontamination”) and after removing contaminants identified using two in silico (“NTConly” and “NTC + decontam”) approaches.

**Additional file 10. **Shifts in OTU-level proportions prior to and following the removal of “potential contaminants” using two in silico approaches for contaminant identification. Per specimen shifts (*n* = 148) in bacterial proportions are shown for eight OTUs classified as four genera A) *Staphylococcus*, B) *Streptococcus*, C) *Acinetobacter* and D) *Stenotrophomonas*. Open circles and smoothing splines (representing a factor of 2x the standard deviation) denote bacterial proportions (Y-axis) for each of the specimens (X-axis). Red: Proportions prior to decontamination; Blue: Proportions following the removal of “potential contaminants” identified using the “NTConly” approach; Yellow: Proportions following the removal of “potential contaminants” identified using the “NTC + decontam” approach.

## Data Availability

The dataset supporting the conclusions of this article is available in the National Center for Biotechnology Information (NCBI) Sequence Read Archive (SRA) under the BioProject ID PRJNA548658, BioSamples SAMN12045520 to SAMN12045810.

## Abbreviations

16S rRNA: 16S ribosomal ribonucleic acid
ANOVA: Analysis of variance
DCHS: Drakenstein Child Health Study
DNA: Deoxyribonucleic acid
IQR: Interquartile range
Ilr: Isometric logratio transformation
IS: Induced sputum
Kit-QS: DSP Virus/Pathogen Mini Kit®
Kit-ZB: ZymoBIOMICS DNA Miniprep Kit
Log-ratio: Logarithm of ratio-transformed data
NP: Nasopharyngeal
NTC: No template control
OTU: Operational taxonomic unit
PCR: Polymerase chain reaction
PERMANOVA: Permutational Multivariate Analysis of Variance
Primestore: PrimeStore® Molecular Transport medium
R^2^: coefficient of determination in linear regression analysis
STGG: Skim-milk, Tryptone, Glucose and Glycerol

## References

[1] Hong KH, Hong SK, Cho SI, Ra E, Han KH, Kang SB (2016). Analysis of the vaginal microbiome by next-generation sequencing and evaluation of its performance as a clinical diagnostic tool in vaginitis. Ann Lab Med.

[2] Botterel F, Angebault C, Cabaret O, Stressmann FA, Costa JM, Wallet F (2018). Fungal and bacterial diversity of airway microbiota in adults with cystic fibrosis: concordance between conventional methods and ultra-deep sequencing, and their practical use in the clinical laboratory. Mycopathologia..

[3] Wang H, Altemus J, Niazi F, Green H, Calhoun BC, Sturgis C (2017). Breast tissue, oral and urinary microbiomes in breast cancer. Oncotarget..

[4] Tropini C, Earle KA, Huang KC, Sonnenburg JL (2017). The Gut Microbiome: Connecting Spatial Organization to Function. Cell Host Microbe.

[5] Scheithauer TPM, Dallinga-Thie GM, de Vos WM, Nieuwdorp M, van Raalte DH (2016). Causality of small and large intestinal microbiota in weight regulation and insulin resistance. Mol Metab Elsevier GmbH.

[6] Yatera K, Noguchi S, Mukae H (2018). The microbiome in the lower respiratory tract. Respir Investig.

[7] Dickson R, Erb-Downward J, Huffnagle G (2014). Towards an ecology of the lung: new conceptual models of pulmonary microbiology and pneumonia pathogenesis. Lancet Respir Med.

[8] Dickson RP, Erb-Downward JR, Freeman CM, Mccloskey L, Falkowski NR, Huffnagle GB (2017). Bacterial topography of the healthy human lower respiratory tract. MBio..

[9] Collado MC, Rautava S, Aakko J, Isolauri E, Salminen S (2016). Human gut colonisation may be initiated in utero by distinct microbial communities in the placenta and amniotic fluid. Sci Rep.

[10] Perez-Muñoz ME, Arrieta MC, Ramer-Tait AE, Walter J (2017). A critical assessment of the “sterile womb” and “in utero colonization” hypotheses: implications for research on the pioneer infant microbiome. Microbiome.

[11] Pelzer E, Gomez-Arango LF, Barrett HL, Nitert MD (2017). Review: maternal health and the placental microbiome. Placenta.

[12] Kuperman AA, Zimmerman A, Hamadia S, Ziv O, Gurevich V, Fichtman B (2020). Deep microbial analysis of multiple placentas shows no evidence for a placental microbiome. BJOG.

[13] Drengenes C, Wiker HG, Kalananthan T, Nordeide E, Eagan TML, Nielsen R (2019). Laboratory contamination in airway microbiome studies. BMC Microbiol.

[14] Dahlberg J, Sun L, Waller KP, Ostensson K, Mcguire M, Agenas S (2019). Microbiota data from low biomass milk samples is markedly affected by laboratory and reagent contamination. PLoS One.

[15] Marsh RL, Nelson MT, Pope CE, Leach AJ, Hoffman LR, Chang AB (2018). How low can we go? The implications of low bacterial load in respiratory microbiota studies. Pneumonia..

[16] Lauder AP, Roche AM, Sherrill-Mix S, Bailey A, Laughlin AL, Bittinger K (2016). Comparison of placenta samples with contamination controls does not provide evidence for a distinct placenta microbiota. Microbiome.

[17] Biesbroek G, Sanders EAM, Roeselers G, Wang X, Caspers MPM, Trzciński K, et al. Deep sequencing analyses of low density microbial communities: working at the boundary of accurate microbiota detection. PLoS One. 2012;7:e32942. 10.1371/journal.pone.0032942 PMC3295791 22412957

[18] Salter SJ, Cox MJ, Turek EM, Calus ST, Cookson WO, Moffatt MF (2014). Reagent and laboratory contamination can critically impact sequence-based microbiome analyses. BMC Biol.

[19] Kim D, Hofstaedter CE, Zhao C, Mattei L, Tanes C, Clarke E (2017). Optimizing methods and dodging pitfalls in microbiome research. Microbiome.

[20] Minich JJ, Sanders JG, Amir A, Humphrey G, Gilbert JA, Knight R (2019). Quantifying and Understanding Well-to-Well Contamination in Microbiome Research. mSystems.

[21] Jervis-Bardy J, Leong LEX, Marri S, Smith RJ, Choo JM, Smith-Vaughan HC (2015). Deriving accurate microbiota profiles from human samples with low bacterial content through post-sequencing processing of Illumina MiSeq data. Microbiome..

[22] Barton HA, Taylor NM, Lubbers BR, Pemberton AC (2006). DNA extraction from low-biomass carbonate rock: an improved method with reduced contamination and the low-biomass contaminant database. J Microbiol Methods.

[23] Pollock J, Glendinning L, Wisedchanwet T, Watson M (2018). The madness of microbiome: attempting to find consensus “Best practice” for 16S microbiome studies. Appl Environ Microbiol.

[24] Hiergeist A, Reischl U (2016). Multicenter quality assessment of 16S ribosomal DNA-sequencing for microbiome analyses reveals high inter-center variability. Int J Med Microbiol.

[25] Man WH, De Steenhuijsen Piters WAA, Bogaert D (2017). The microbiota of the respiratory tract: gatekeeper to respiratory health. Nat Rev Microbiol.

[26] Zar HJ, Barnett W, Myer L, Stein DJ, Nicol MP (2014). Investigating the early-life determinants of illness in Africa: the Drakenstein child health study. Thorax..

[27] Cabral DJ, Wurster JI, Flokas ME, Alevizakos M, Zabat M, Korry BJ (2017). The salivary microbiome is consistent between subjects and resistant to impacts of short-term hospitalization. Sci Rep.

[28] Grønseth R, Drengenes C, Wiker HG, Tangedal S, Xue Y, Husebø GR (2017). Protected sampling is preferable in bronchoscopic studies of the airway microbiome. ERJ Open Res.

[29] Davis NM, Proctor D, Holmes SP, Relman DA, Callahan BJ. Simple statistical identification and removal of contaminant sequences in marker-gene and metagenomics data. Microbiome. 2018;6:226. 10.1186/s40168-018-0605-2 PMC6298009 30558668

[30] Eisenhofer R, Minich JJ, Marotz C, Cooper A, Knight R, Weyrich LS. Contamination in low microbial biomass microbiome studies: issues and recommendations. Trends Microbiol. 2019;27:105–17. 10.1016/j.tim.2018.11.003 30497919

[31] Adegoke AA, Stenström TA, Okoh AI (2017). Stenotrophomonas maltophilia as an emerging ubiquitous pathogen: looking beyond contemporary antibiotic therapy. Front Microbiol.

[32] Yamada K, Yanagihara K, Araki N, Harada Y, Morinaga Y, Akamatsu N (2012). Clinical characteristics of tertiary hospital patients from whom Acinetobacter calcoaceticus-Acinetobacter baumannii Complex strains were isolated. Intern Med.

[33] Silvia Munoz-Price L, Weinstein RA (2012). Acinetobacter infection. N Engl J Med.

[34] Theis KR, Romero R, Winters AD, Greenberg JM, Gomez-Lopez N, Alhousseini A (2019). Does the human placenta delivered at term have a microbiota? Results of cultivation, quantitative real-time PCR, 16S rRNA gene sequencing, and metagenomics. Am J Obstet Gynecol.

[35] Willner D, Daly J, Whiley D, Grimwood K, Wainwright CE, Hugenholtz P (2012). Comparison of DNA extraction methods for microbial community profiling with an application to pediatric bronchoalveolar lavage samples. PLoS One.

[36] Abusleme L, Hong B-Y, Dupuy AK, Strausbaugh LD, Diaz PI (2014). Influence of DNA extraction on oral microbial profiles obtained via 16S rRNA gene sequencing. J Oral Microbiol.

[37] Douglas CA, Ivey KL, Papanicolas LE, Best KP, Muhlhausler BS, Rogers GB (2020). DNA extraction approaches substantially influence the assessment of the human breast milk microbiome. Sci Rep.

[38] Velásquez-Mejía EP, de la Cuesta-Zuluaga J, Escobar JS. Impact of DNA extraction, sample dilution, and reagent contamination on 16S rRNA gene sequencing of human feces. Appl Microbiol Biotechnol. 2018;102:403–11. 10.1007/s00253-017-8583-z 29079861

[39] Schloss PD, Gevers D, Westcott SL. Reducing the effects of PCR amplification and sequencing artifacts on 16s rRNA-based studies. PLoS One. 2011;6:e27310. 10.1371/journal.pone.0027310 PMC3237409 22194782

[40] Gibbons SM, Duvallet C, Alm EJ (2018). Correcting for batch effects in case-control microbiome studies. PLoS Comput Biol.

[41] Fouhy F, Clooney AG, Stanton C, Claesson MJ, Cotter PD (2016). 16S rRNA gene sequencing of mock microbial populations-impact of DNA extraction method, primer choice and sequencing platform. BMC Microbiol.

[42] D’Amore R, Ijaz UZ, Schirmer M, Kenny JG, Gregory R, Darby AC (2016). A comprehensive benchmarking study of protocols and sequencing platforms for 16S rRNA community profiling. BMC Genomics.

[43] Brooks JP, Edwards DJ, Harwich MD, Rivera MC, Fettweis JM, Serrano MG (2015). The truth about metagenomics: quantifying and counteracting bias in 16S rRNA studies ecological and evolutionary microbiology. BMC Microbiol.

[44] Salipante SJ, Kawashima T, Rosenthal C, Hoogestraat DR, Cummings LA, Sengupta DJ (2014). Performance comparison of Illumina and ion torrent next-generation sequencing platforms for 16S rRNA-based bacterial community profiling. Appl Environ Microbiol.

[45] Laursen MF, Dalgaard MD, Bahl MI (2017). Genomic GC-content affects the accuracy of 16S rRNA gene sequencing bsed microbial profiling due to PCR bias. Front Microbiol.

[46] Schneeberger PHH, Prescod J, Levy L, Hwang D, Martinu T, Coburn B (2019). Microbiota analysis optimization for human bronchoalveolar lavage fluid. Microbiome.

[47] Bogaert D, Keijser B, Huse S, Rossen J, Veenhoven R, van Gils E, et al. Variability and diversity of nasopharyngeal microbiota in children: a metagenomic analysis. PLoS One. 2011;6:e17035. 10.1371/journal.pone.0017035 PMC3046172 21386965

[48] Guo F, Zhang T (2013). Biases during DNA extraction of activated sludge samples revealed by high throughput sequencing. Appl Microbiol Biotechnol.

[49] Mirsepasi H, Persson S, Struve C, Andersen LOB, Petersen AM, Krogfelt KA. Microbial diversity in fecal samples depends on DNA extraction method: EasyMag DNA extraction compared to QIAamp DNA stool mini kit extraction. BMC Res Notes. 2014;7:50. 10.1186/1756-0500-7-50 PMC4015497 24447346

[50] Nakayama Y, Yamaguchi H, Einaga N, Esumi M. Pitfalls of DNA quantification using DNA-binding fluorescent dyes and suggested solutions. PLoS One. 2016;11:1–12. 10.1371/journal.pone.0150528 PMC4777359 26937682

[51] Luhung I, Wu Y, Ng CK, Miller D, Cao B, Chang VWC (2015). Protocol improvements for low concentration DNA-based bioaerosol sampling and analysis. PLoS One.

[52] Hussing C, Kampmann ML, Mogensen HS, Børsting C, Morling N (2018). Quantification of massively parallel sequencing libraries - a comparative study of eight methods. Sci Rep.

[53] Stinson LF, Keelan JA, Payne MS (2019). Identification and removal of contaminating microbial DNA from PCR reagents: impact on low-biomass microbiome analyses. Lett Appl Microbiol.

[54] Stinson L, Boyce M, Payne M, Keelan J (2019). The not-so-sterile womb: evidence that the human fetus is exposed to bacteria prior to birth. Front Microbiol.

[55] Leon LJ, Doyle R, Diez-Benavente E, Clark TG, Klein N, Stanier P (2018). Enrichment of clinically relevant organisms in spontaneous preterm-delivered placentas and reagent contamination across all clinical groups in a large pregnancy cohort in the United Kingdom. Appl Environ Microbiol.

[56] Kulakov LA, Mcalister MB, Ogden KL, Larkin MJ, Hanlon JFO (2002). Analysis of Bacteria contaminating ultrapure water in industrial systems analysis of Bacteria contaminating ultrapure water in industrial systems. Appl Environ Microbiol.

[57] Kéki Z, Grébner K, Bohus V, Márialigeti K, Tóth EM (2013). Application of special oligotrophic media for cultivation of bacterial communities originated from ultrapure water. Acta Microbiol Immunol Hung.

[58] Glassing A, Dowd SE, Galandiuk S, Davis B, Chiodini RJ (2016). Inherent bacterial DNA contamination of extraction and sequencing reagents may affect interpretation of microbiota in low bacterial biomass samples. Gut Pathog.

[59] Lazarevic V, Gaïa N, Girard M, Schrenzel J (2016). Decontamination of 16S rRNA gene amplicon sequence datasets based on bacterial load assessment by qPCR. BMC Microbiol.

[60] Callahan BJ, DiGiulio DB, Goltsman DSA, Sun CL, Costello EK, Jeganathan P (2017). Replication and refinement of a vaginal microbial signature of preterm birth in two racially distinct cohorts of US women. Proc Natl Acad Sci.

[61] Aslanzadeh J, Preventing PCR (2004). Amplification carryover contamination in a clinical laboratory. Ann Clin Lab Sci.

[62] Boyle A, Timoney J, Newton J, Hines M, Waller A, Buchanan B (2018). Streptococcus equi infections in horses: guidelines for treatment, control, and prevention of strangles—revised consensus statement. J Vet Intern Med.

[63] Noguchi S, Yatera K, Kawanami T, Yamasaki K, Naito K, Akata K (2015). The clinical features of respiratory infections caused by the Streptococcus anginosus group. BMC Pulm Med.

[64] Daum LT, Worthy SA, Yim KC, Nogueras M, Schuman RF, Choi YW (2011). A clinical specimen collection and transport medium for molecular diagnostic and genomic applications. Epidemiol Infect.

[65] Zar H, Barnett W, Stadler A, Gardner-Lubbe S, Myer L, Nicol M (2016). Aetiology of childhood pneumonia in a well vaccinated south African birth cohort: a nested case-control study. Lancet Respir Med.

[66] Satzke C, Turner P, Virolainen-Julkunen A, Adrian PV, Antonio M, Hare KM (2013). Standard method for detecting upper respiratory carriage of Streptococcus pneumoniae: updated recommendations from the World Health Organization pneumococcal carriage working group. Vaccine..

[67] Salter SJ, Turner C, Watthanaworawit W, de Goffau MC, Wagner J, Parkhill J (2017). A longitudinal study of the infant nasopharyngeal microbiota: the effects of age, illness and antibiotic use in a cohort of south east Asian children. PLoS Negl Trop Dis.

[68] Claassen-Weitz S, Gardner-Lubbe S, Nicol P, Botha G, Mounaud S, Shankar J (2018). HIV-exposure, early life feeding practices and delivery mode impacts on faecal bacterial profiles in a south African birth cohort. Sci Rep.

[69] Caporaso JG, Lauber CL, Walters WA, Berg-lyons D, Lozupone CA, Turnbaugh PJ (2010). Global patterns of 16S rRNA diversity at a depth of millions of sequences per sample. PNAS..

[70] Illumina Proprietary (2013). MiSeq ® Reagent Kit v3 Reagent Preparation Guide.

[71] Illumina Proprietary (2014). MiSeq ® System User Guide.

[72] Andrews S (2010). FastQC: a quality control tool for high throughput sequence data.

[73] Ewels P, Magnusson M, Lundin S, Käller M (2016). MultiQC: summarize analysis results for multiple tools and samples in a single report. Bioinformatics..

[74] Edgar RC (2013). UPARSE: highly accurate OTU sequences from microbial amplicon reads. Nat Methods.

[75] Edgar R. Search and clustering orders of magnitude faster than BLAST. Bioinformatics. 2010;26:2460–1. 10.1093/bioinformatics/btq461 20709691

[76] Caporaso JG, Kuczynski J, Stombaugh J, Bittinger K, Bushman FD, Costello EK (2010). QIIME allows analysis of high-throughput community sequencing data. Nat Methods.

[77] Pruesse E, Quast C, Knittel K, Fuchs BM, Ludwig W, Peplies J (2007). SILVA: a comprehensive online resource for quality checked and aligned ribosomal RNA sequence data compatible with ARB. Nucleic Acids Res.

[78] Quast C, Pruesse E, Yilmaz P, Gerken J, Schweer T, Yarza P (2013). The SILVA ribosomal RNA gene database project: improved data processing and web-based tools. Nucleic Acids Res.

[79] Wang Q, Garrity GM, Tiedje JM, Cole JR (2007). Naive Bayesian classifier for rapid assignment of rRNA sequences into the new bacterial taxonomy. Appl Environ Microbiol.

[80] Di Tommaso P, Chatzou M, Floden EW, Barja PP, Palumbo E, Notredame C (2017). Nextflow enables reproducible computational workflows. Nat Biotechnol.

[81] R Core Team. R: A language and environment for statistical computing.Vienna:R Foundation for Statistical Computing, 2018. Online: https://www.R-project.org/.

[82] Anders S, Huber W (2010). Differential expression analysis for sequence count data. Genome Biol.

[83] McMurdie PJ, Holmes S (2014). Waste not, want not: why rarefying microbiome data is inadmissible. PLoS Comput Biol.

[84] Fernandes AD, Reid JN, Macklaim JM, McMurrough TA, Edgell DR, Gloor GB (2014). Unifying the analysis of high-throughput sequencing datasets: characterizing RNA-seq, 16S rRNA gene sequencing and selective growth experiments by compositional data analysis. Microbiome..

[85] Oksanen J, Blanchet F, Kindt R, Legendre P, Minchin P, O’Hara R, et al. Vegan: Community Ecology Package. 2013.

[86] Templ M, Hron K, Filzmoser P. robCompositions: An R-package for Robust Statistical Analysis of Compositional Data. Compos Data Anal Theory Appl. Chichester: Wiley; 2011:341–55.

[87] Shannon CE (1948). A mathematical theory of communication. Bell Syst Tech J.

[88] Gower J, Lubbe S, Le Roux N (2011). Understanding Biplots.

[89] Martin-Fernandez J, Palarea-Albaladejo J, Olea R. Dealing with Zeros. In: Pawlowsky-Glahn V, Buccianti A, editors. Compos Data Anal Theory Appl. Chichester: Wiley; 2011:43–58.

[90] Aitchison J (1982). The statistical analysis of compositional data. J R Stat Soc.

[91] Aitchison J, Greenacre M (2002). Biplots of compositional data. J R Stat Soc: Ser C: Appl Stat.

[92] Morgan XC, Huttenhower C (2012). Chapter 12: human microbiome analysis. PLoS Comput Biol.

[93] Faith DP, Minchin PR, Belbin L (1987). Compositional dissimilarity as a robust measure of ecological distance. Vegetatio..

[94] Bray JR, Curtis JT (1957). An ordination of the upland Forest communities of southern Wisconsin. Ecol Monogr.

[95] Clarke KR, Warwick RM (2001). Change in marine communities: an approach to statistical analysis and interpretation. Second edi.

[96] Draper N, Smith H. Applied regression analysis. Second edi. New York: Wiley; 1981.

