# Supplementary figures and images for: Optimizing 16S rRNA gene profile analysis from low biomass nasopharyngeal and induced sputum specimens
Source: BMC Microbiol. 2020 May 12;20:113. doi: 10.1186/s12866-020-01795-7 (PMC7218582; doi:10.1186/s12866-020-01795-7)

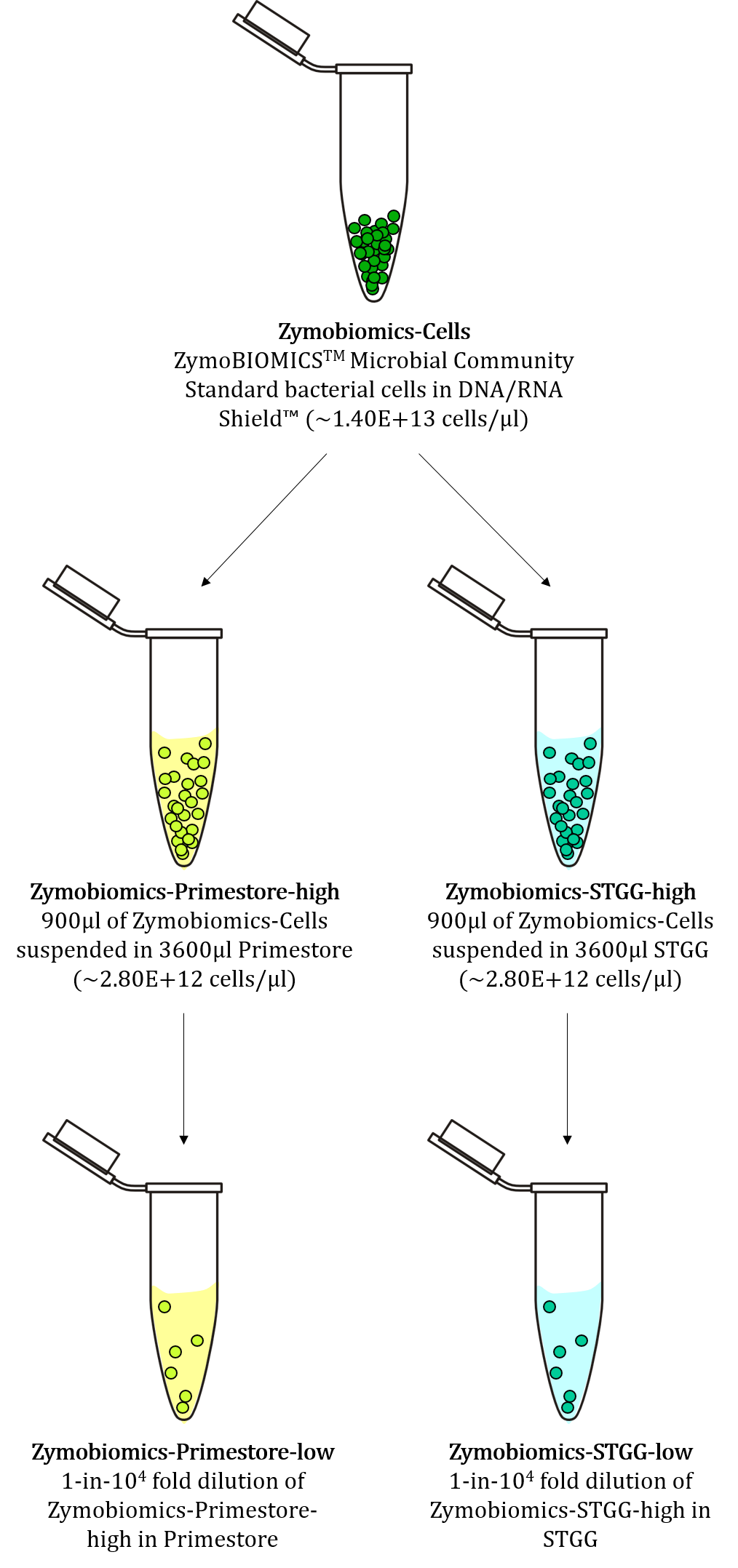

Supplement: Supplementary file 1 — Additional file 1. Four bacterial mock communities used to evaluate the effect of DNA extraction methods, storage buffers and bacterial biomass on 16S rRNA gene sequencing profiles. Zymobiomics-Primestore-high and Zymobiomics-Primestore-low: ZymoBIOMICS™ Microbial Community Standard bacterial cells in DNA/RNA Shield™ (Zymobiomics-Cells) suspended in PrimeStore® Molecular Transport medium (Primestore); Zymobiomics-STGG-high and Zymobiomics-STGG-low: Zymobiomics-Cells suspended in skim-milk tryptone glucose glycerol transport medium (STGG). [file 12866_2020_1795_MOESM1_ESM.tif]

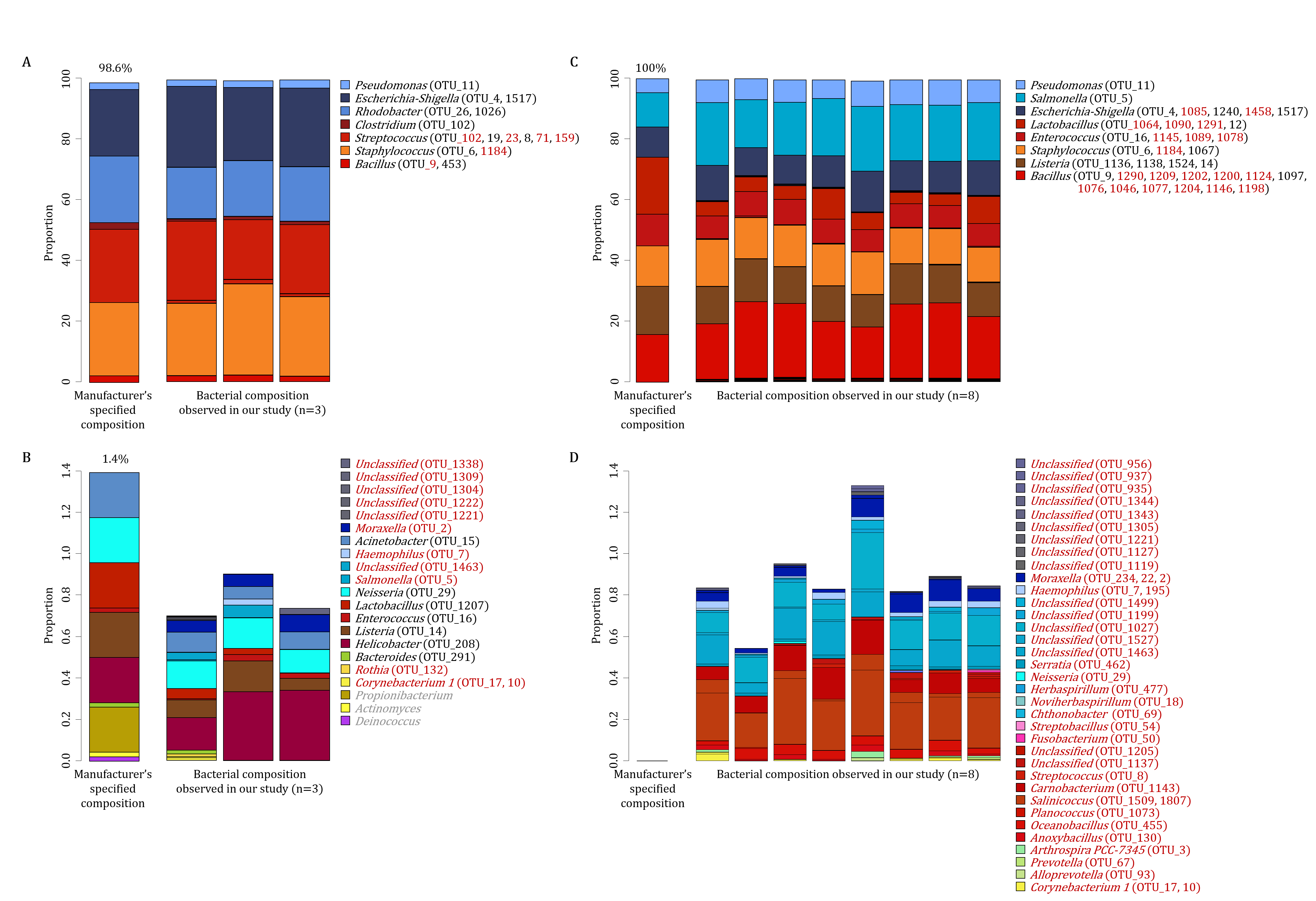

Supplement: Supplementary file 2 — Additional file 2. Operational taxonomic units (OTUs) sequenced from bacterial mock community DNA controls. Manufacturers’ specified versus observed OTU composition from BEI-DNA (A-B) and Zymobiomics-DNA (n = 8) (C-D) mock controls. Panels A and C represent OTUs from bacterial genera detected at mean proportions of > 0.5% from BEI-DNA (A) and Zymobiomics-DNA (C), respectively. Panels B and D represent OTUs from bacterial genera detected at mean proportions of < 0.5% from BEI-DNA (B) and Zymobiomics-DNA (D), respectively. Bacterial genera in grey font are expected in the bacterial mock community DNA controls but missing from the profiles generated in our laboratory. Bacterial genera in red font are not expected in mock community DNA. OTUs in red font are not expected in mock community DNA or unclassifiable at species-level. Bacterial genera are colour-coded according to the phylum to which they belong (Shades of red: Firmicutes; shades of blue: Proteobacteria; shades of yellow: Actinobacteria; olivegreen: Bacteroidetes; seagreen: Cyanobacteria; purple: Deinococcus-Thermus and grey: unclassified). [file 12866_2020_1795_MOESM2_ESM.tif]

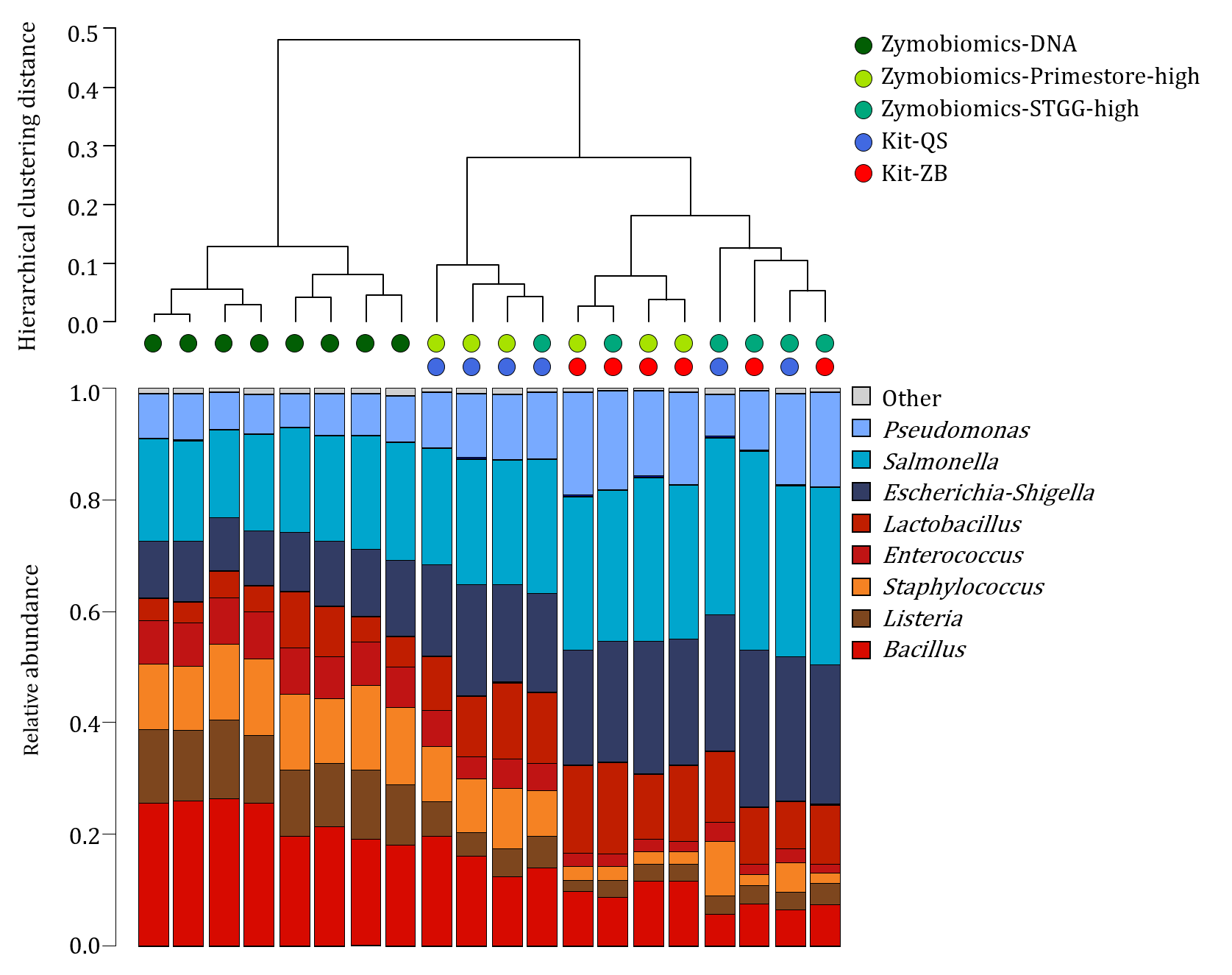

Supplement: Supplementary file 3 — Additional file 3. Bacterial composition of Zymobiomics-DNA (n = 8) compared to Zymobiomics-Primestore-high (n = 6) and Zymobiomics-STGG-high (n = 6). The two high biomass mock communities, Zymobiomics-Primestore-high and Zymobiomics-STGG-high, represent triplicate extractions using two extraction methods (blue filled circles: Kit-QS and red filled circles: Kit-ZB). Zymobiomics-DNA (darkgreen filled circles) were included to validate sequencing profiles generated using the two extraction methods. Unsupervised hierarchical clustering distances are based on Bray Curtis dissimilarity indices calculated at OTU-level. Differences between bacterial mock controls are shown at genus-level, with colour-codes representing phylum-level classification (Shades of blue: Proteobacteria, shades of red: Firmicutes). Genera with proportions < 1% in each of the specimens are grouped together as “Other” and shown in grey. [file 12866_2020_1795_MOESM3_ESM.tif]

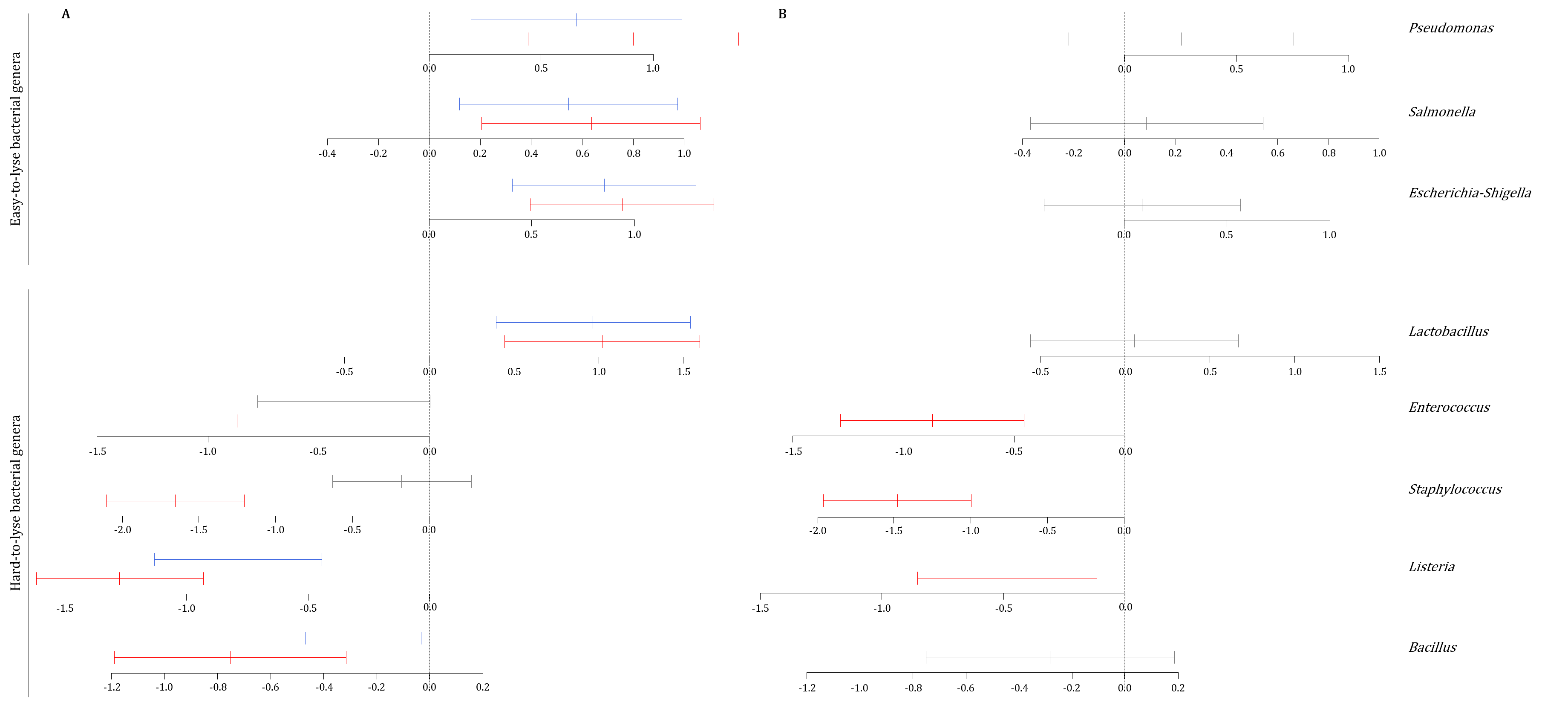

Supplement: Supplementary file 4 — Additional file 4. Differences between hard-and-easy to lyse bacterial profiles from Zymobiomics-DNA (n = 8) and extracts from high bacterial mock community controls [Zymobiomics-Primestore-high (n = 6) and Zymobiomics-STGG-high (n = 6)] using Kit-QS and Kit-ZB. A) The Tukey Honest Significant Difference simultaneous confidence intervals calculated at OTU-level indicate whether bacterial profiles extracted from high biomass bacterial mock community controls using Kit-QS and Kit-ZB differ significantly from Zymobiomics-DNA, and B) between Kit-QS and Kit-ZB. Confidence intervals computed on the isometric logratio transformation (ilr) scale indicates statistical significance at a 5% significance level when it excludes zero. Blue confidence intervals: significant findings for Kit-QS; Red confidence intervals: significant findings for Kit-ZB. [file 12866_2020_1795_MOESM4_ESM.tif]

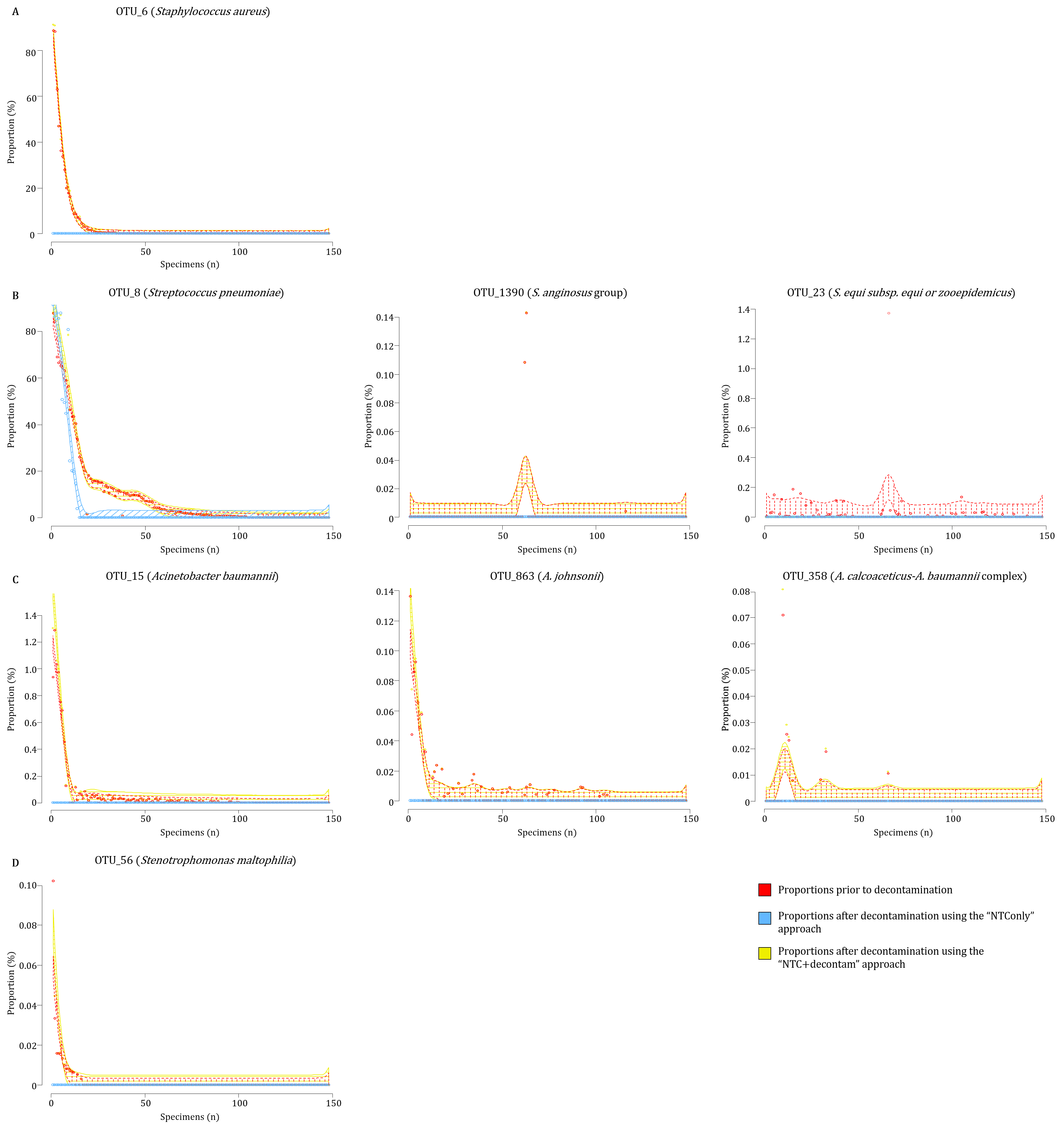

Supplement: Supplementary file 10 — Additional file 10. Shifts in OTU-level proportions prior to and following the removal of “potential contaminants” using two in silico approaches for contaminant identification. Per specimen shifts (n = 148) in bacterial proportions are shown for eight OTUs classified as four genera A) Staphylococcus, B) Streptococcus, C) Acinetobacter and D) Stenotrophomonas. Open circles and smoothing splines (representing a factor of 2x the standard deviation) denote bacterial proportions (Y-axis) for each of the specimens (X-axis). Red: Proportions prior to decontamination; Blue: Proportions following the removal of “potential contaminants” identified using the “NTConly” approach; Yellow: Proportions following the removal of “potential contaminants” identified using the “NTC + decontam” approach. [file 12866_2020_1795_MOESM10_ESM.tif]
