# Supplementary material for: Optimizing 16S rRNA gene profile analysis from low biomass nasopharyngeal and induced sputum specimens
Source: BMC Microbiol. 2020 May 12;20:113. doi: 10.1186/s12866-020-01795-7 (PMC7218582; doi:10.1186/s12866-020-01795-7)
Supplement: Supplementary file 5 — Additional file 5. Sequencing output from technical repeats (n = 209) stratified by 16S rRNA gene copy numbers and participant age at specimen collection. [file 12866_2020_1795_MOESM5_ESM.docx]

**Additional file 5.** Sequencing output from technical repeats (n=209) stratified by 16S rRNA gene copy numbers and participant age at specimen collection

|  | 16S rRNA gene copies/µl: < 500 | | | | |  | 16S rRNA gene copies/µl: > 500 | | | | |
| --- | --- | --- | --- | --- | --- | --- | --- | --- | --- | --- | --- |
|  | < 0 days | > 0-14 days | > 14-30 days | > 30-60 days | > 60 days |  | < 0 days | > 0-14 days | > 14-30 days | > 30-60 days | > 60 days |
| Number of specimens (%) | 26  (42.6) | 11  (18.0) | 3  (4.9) | 3  (4.9) | 18  (29.5) |  | - | 4  (2.7) | 12  (8.1) | 13  (8.8) | 119  (80.4) |
| Median read counts following  bioinformatic processes (IQR) | 2932  (982-5597) | 1890  (1000-4451) | 9315  (8017-12198) | 2042  (1563-5867) | 9972  (4232-21009) |  | - | 6610  (5981-7680) | 10158  (4216-21832) | 14650  (10350-16947) | 13657  (7091-20382) |
| Median Shannon diversity indices (alpha diversity) (IQR) | 2.8  (2.1-3.1) | 2.9  (2.7-3.1) | 0.8  (0.5-1.6) | 2.7  (2.6-2.8) | 1.7  (1.3-2.5) |  | - | 1.7  (1.6-1.8) | 0.8  (0.4-1.0) | 0.6  (0.5-0.8) | 0.8  (0.6-1.1) |
| Median OTU counts (IQR) | 101  (43-133) | 118  (98-143) | 57  (38-119) | 101  (83-133) | 93  (44-153) |  | - | 145  (135-154) | 31  (14-61) | 73  (38-133) | 42  (26-59) |
| Per specimen frequency of spurious OTUs (spurious OTUs per age category/biological repeats per category) | 3.5  (90/26) | 3.6  (39/11) | 4.3  (13/3) | 2  (6/3) | 2.3  (42/18) |  | - | 2.5  (10/4) | 0.8  (10/12) | 2.3  (30/13) | 0.8  (95/119) |

*IQR: Interquartile range*

*OTU: Operational taxonomic unit*
