# Supplementary material for: Optimizing 16S rRNA gene profile analysis from low biomass nasopharyngeal and induced sputum specimens
Source: BMC Microbiol. 2020 May 12;20:113. doi: 10.1186/s12866-020-01795-7 (PMC7218582; doi:10.1186/s12866-020-01795-7)
Supplement: Supplementary file 6 — Additional file 6. Sequencing reproducibility is associated with participant age at specimen collection, 16S rRNA gene copy numbers and read counts. [file 12866_2020_1795_MOESM6_ESM.docx]

**Additional file 6.** Sequencing reproducibility is associated with participant age at specimen collection, 16S rRNA gene copy numbers and read counts

|  | Number of specimens with  R^2^ < 0.90 (%) | |  | Number of specimens with  R^2^ > 0.90 (%) | |
| --- | --- | --- | --- | --- | --- |
| Total | 22 (14.3) | |  | 132 (85.7) | |
| Participant age at specimen collection (days) |  | |  |  | |
| < 7 | 10 (45.5) | |  | 18 (13.6) | |
| < 14 | 11 (50.0) | |  | 23 (17.4) | |
| < 30 | 13 (59.0) | |  | 30 (22.7) | |
| < 60 | 18 (81.8) | |  | 37 (28.0) | |
| 16S rRNA gene copy numbers (copies/µl) |  |  |  |  |  |
| < 100 | 11 (50.0)* | 11 (50.0)* |  | 20 (15.2)* | 15 (11.4)* |
| < 500 | 17 (77.3)* | 17 (77.3)* |  | 34 (25.8)* | 36 (27.3)* |
| < 1000 | 17 (77.3)* | 19 (86.4)* |  | 36 (27.3)* | 41 (31.1)* |
| Read counts (following bioinformatic processes) |  |  |  |  |  |
| < 2000 | 12 (54.5)* | 5 (22.7)* |  | 9 (6.8)* | 6 (4.6)* |
| < 4000 | 13 (59.0)* | 10 (45.5)* |  | 24 (18.2)* | 14 (10.6)* |
| < 6000 | 15 (68.2)* | 13 (59.0)* |  | 41 (31.1)* | 38 (28.8)* |
| < 8000 | 16 (72.7)* | 15 (68.2)* |  | 49 (37.1)* | 44 (33.3)* |
| < 10000 | 18 (81.8)* | 18 (81.8)* |  | 58 (43.9)* | 51 (38.6)* |

**When investigating associations between 16S rRNA gene copy numbers and reproducibility, we considered two 16S rRNA gene copy numbers (from each technical repeat) for each R^2^ value calculated. The same applied for read counts.*
